# Genome-Wide Comparative Analysis of Chemosensory Gene Families in Five Tsetse Fly Species

**DOI:** 10.1371/journal.pntd.0004421

**Published:** 2016-02-17

**Authors:** Rosaline Macharia, Paul Mireji, Edwin Murungi, Grace Murilla, Alan Christoffels, Serap Aksoy, Daniel Masiga

**Affiliations:** 1 Molecular Biology and Bioinformatics Unit, International Centre of Insect Physiology and Ecology, Nairobi, Kenya; 2 South African National Bioinformatics Institute, University of the Western Cape, Cape Town, South Africa; 3 Department of Epidemiology of Microbial Diseases, Yale School of Public Heath, New Haven, Connecticut, United States of America; 4 Biotechnology Research Institute, Kenya Agricultural and Livestock Research Organization, Kikuyu, Kenya; 5 Department of Biochemistry and Molecular Biology, Egerton University, Njoro, Kenya; Liverpool School of Tropical Medicine, UNITED KINGDOM

## Abstract

For decades, odour-baited traps have been used for control of tsetse flies (Diptera; Glossinidae), vectors of African trypanosomes. However, differential responses to known attractants have been reported in different *Glossina* species, hindering establishment of a universal vector control tool. Availability of full genome sequences of five *Glossina* species offers an opportunity to compare their chemosensory repertoire and enhance our understanding of their biology in relation to chemosensation. Here, we identified and annotated the major chemosensory gene families in *Glossina*. We identified a total of 118, 115, 124, and 123 chemosensory genes in *Glossina austeni*, *G*. *brevipalpis*, *G*. *f*. *fuscipes*, *G*. *pallidipes*, respectively, relative to 127 reported in *G*. *m*. *morsitans*. Our results show that tsetse fly genomes have fewer chemosensory genes when compared to other dipterans such as *Musca domestica* (*n*>393), *Drosophila melanogaster* (*n* = 246) and *Anopheles gambiae* (*n*>247). We also found that *Glossina* chemosensory genes are dispersed across distantly located scaffolds in their respective genomes, in contrast to other insects like *D*. *melanogaster* whose genes occur in clusters. Further, *Glossina* appears to be devoid of sugar receptors and to have expanded CO_2_ associated receptors, potentially reflecting *Glossina*'s obligate hematophagy and the need to detect hosts that may be out of sight. We also identified, in all species, homologs of Ir84a; a *Drosophila*-specific ionotropic receptor that promotes male courtship suggesting that this is a conserved trait in tsetse flies. Notably, our selection analysis revealed that a total of four gene loci (Gr21a, GluRIIA, Gr28b, and Obp83a) were under positive selection, which confers fitness advantage to species. These findings provide a platform for studies to further define the language of communication of tsetse with their environment, and influence development of novel approaches for control.

## Author Summary

Chemical sensing is crucial to survival of tsetse flies; the sole cyclical vectors of African trypanosomes that cause the neglected zoonotic tropical disease sleeping sickness in humans. For many years, vector control has been used to mitigate trypanosome infections among rural populations of sub-Saharan Africa. Nevertheless, development of an all-inclusive strategy to control tsetse flies using odour-baited traps has been limited by disparate responses to the odors exhibited by various tsetse species. In this study, proteins that are putatively involved in chemical sensing were identified and compared among five tsetse species and their close relatives with an aim of enhancing our knowledge on tsetse olfaction. Our findings suggest that the chemosensory genes are conserved across tsetse fly species despite their documented differential responses in odours. We found no species-specific sequence variations among the five species to suggest that differential response to odours is due to loss or gain of genes. It could therefore be hypothesized that the observed differences emerge during the downstream processing of odour molecules involving post translational modification of the chemosensory proteins. We thus recommend functional studies on the identified proteins to determine their roles and molecular interactions.

## Introduction

Tsetse flies (*Glossina* spp.) are the sole cyclical vectors of African trypanosomes that cause the devastating Human African Trypanosomiasis (HAT, sleeping sickness) and Animal African Trypanosomiasis (AAT, nagana) across sub-Saharan Africa [1]. It is estimated that approximately 70 million people and 50 million cattle inhabiting tsetse-fly infested areas are at risk of contracting trypanosomiasis [2,3], and that nagana accounts for up to $ 4.75 billion annual losses [4]. Currently, there are no prophylactic drugs or vaccines against HAT. Moreover, the available chemotherapeutic remedies are not ideal due to their toxicity, difficulty in administration and growing resistance [4–6].

It has long been known that comprehensive and sustainable control of trypanosomiasis requires a vector control component [7]. Efforts to suppress tsetse populations include trapping, which rely on traps baited with various host derived odours [8–10]. Differences in response to the available baits have been observed among tsetse species and/or between males and female flies [11,12]. For example the palpalis/riverine species are thought to be attracted to kairomones released by monitor lizards, but unresponsive to odours that are highly attractive to Savannah species [13]. This differentiation of responses to odours is shown by the varied host preference in different sub-groups [14,15].

Chemoreception in tsetse and other insects is mediated by a group of peri-receptor and surface proteins/receptors encoded by different gene families [16] including: odorant binding proteins (OBPs), chemosensory proteins (CSPs), sensory neuron membrane proteins (SNMPs), gustatory receptors (GRs), ionotropic receptors (IRs) and odorant receptors (ORs). Genes encoding various chemosensory proteins are expressed at different olfactory receptor neurons (ORNs) located mainly on the surface of antennae and in fewer numbers on the maxillary palpi [17,18].

The OBPs and CSPs that recognize and solubilize hydrophobic odor molecules, shuttling them to the dendritic membrane [19,20], are characterized by the presence of a signal peptide and α-helices joined by disulphide bonds [21]. OBPs (~150 aa) are highly diverse proteins thought to bind to a wide range of odorants including pheromones. In *Drosophila*, four different sub-groups of OBPs have been described based on the number of conserved cysteine residues that participate in formation of their tertiary structures. These include (i) Classic OBPs that harbor six highly conserved cysteines and three disulphide bridges, (ii) Classic-Dimer OBPs that have two of the six-cysteine signatures, (iii) Minus-C OBPs which have lost two conserved cysteine residues and (iv) Plus-C OBPs which have additional conserved cysteine residues and a conserved proline [22]. On the other hand, CSPs are characterized by four conserved cysteines and an average length of 130 aa [19]. The latter have been implicated in non-olfactory functions in *Drosophila* [23]. Expression of OBPs and CSPs has been linked to host seeking by adult female in *G*. *m*. *morsitans* [24,25]. A third class of proteins that play a role in olfaction is the SNMPs which belong to the CD36 super family that act as scavenger proteins in humans [25–27]. An earlier study by Xa and colleagues demonstrated involvement of SNMP1 in chemoreception as a requirement for pheromone detection by *Drosophila* [28].

Insect ORs are highly diverse and are characterized by a reversed N-terminal topology and presence of a seven trans-membrane domain [29]. Specific ORs combine with Orco (Or83b), a non-conventional co-receptor, to form functional ion channels that confer specificity to a variety of semiochemicals [29,30]. Fewer ORs were identified in *G*. *m*. *morsitans* relative to *D*. *melanogaster* genome, but with an expansion of a gene critical role in recognition of male the pheromone, *cis*-vaccenyl acetate (cVA) (OR67d) [31]. Insect GRs are responsible for distinguishing between odor tastes and contact pheromones [16,32]. Fewer GRs were also identified in tsetse than in *D*. *melanogaster* and other Diptera [28]. No receptors for sugar were identified in *G*. *m*. *morsitans*, probably due to the hematophagous feeding behavior of the insect [31].

Another class of divergent insect chemosensory receptors is the ionotropic receptors; IRs [33,34]. The IRs, like ORs, function in complexes formed by up to three subunits and one or two of co-receptors (Ir25a and Ir8a) [33,35]. However, unlike ORs, IRs are expressed by coeloconic olfactory neurons [33], and show responses to a variety of odours including acids, aldehydes, amines and humidity [36]. Between two and three heterodimers in IRs, similar to those observed in ORs, are required to form functional complexes involved in distinct odor perception [33,37]. Antennal IRs are not similar to ionotropic glutamate receptors (iGluRs), but have higher specificity to volatiles than ORs [33]. Characterization of IRs has not been reported among *Glossina* species to date. Insect chemosensory genes are divergent and evolve through duplication, pseudogenisation and/or deletion incidences [38]. Functional olfactory genes have been reported to be under natural selection in other organisms including humans [39] and *Drosophila* [40]. Positive selection confers a fitness advantage to a given species relative to the rest of the population and/or increases its genetic diversity [36]. On the other hand, negative (purifying) selection is known to remove deleterious alleles [41].

Understanding molecular factors that underpin the differences observed among species of tsetse, in response to odours is key to success of vector control and management of this vector-borne disease. Availability of the complete genome sequences of five *Glossina* genomes presents fortuity for comparing molecular properties of proteins that mediate olfaction at species level. Recent characterization of major chemosensory protein gene families (OBPs and CSPs) [24,25] and identification of genes encoding GRs and ORs in *G*. *m*. *morsitans* [31,42] formed a basis to compare genes in different tsetse species. We hypothesize that differences in responses to odours observed among tsetse species are mediated by differences in their chemosensory repertoire. Genes annotated in four newly sequenced tsetse species were compared with their homologs in *G*. *m*. *morsitans* and close dipterans (*Ceratitis capitata*, *D*. *melanogaster*, *M*. *domestica* and *An*. *gambiae*). The choice of insects used in comparative analysis was informed by their evolutionary grouping under tree of life [43]. Results obtained from this study will form a prototype for undertaking functional studies on tsetse chemosensory proteins to identify their role in tsetse speciation and differential host-selection. Further, the findings will provide insight for improvement of existing vector control tools and development of novel strategies.

## Methods

### Identification and Annotation of Chemosensory Genes

Genome sequences of *G*. *austeni*, *G*. *brevipalpis*, *G*. *f*. *fuscipes* and *G*. *pallidipes*, their associated gene sets (transcripts, peptides) and gene loci feature files were retrieved from VectorBase database, Release VB-2014-12 [44]. Chemosensory gene sequences from *D*. *melanogaster*, *An*. *gambiae*, and *M*. *domestica* were sourced from FlyBase [45], Uniprot [46], and [47] (through Hugh Robertson of University of Illinois), respectively. The OBP sequences for *C*. *capitata* were obtained from GenBank [48] using the published Accession numbers [49]. BLASTp algorithm with an e-value cutoff of ≤ 1.0^e-5^ was used to identify homologs to chemosensory genes annotated in *G*. *m*. *morsitans* [24,31] and/or in *D*. *melanogaster* [16]. Presence of definitive domain(s) in CSPs (OS-D-like), OBPs (PBP/GOBP), ORs (7tm-6), GRs (7tm-7) and IRs (Lig-Chan, ANF, NMDA) was confirmed through Delta Blast searches against the NCBI’s Conserved Domain Database [50]. Where applicable, gene loci that showed incomplete domains and/or had incomplete sequences were manually curated using Artemis genome viewer tool [51]. For curation, flanking regions of the gene loci (in respective scaffolds) were interrogated for Open Reading Frames (ORF) using NCBI’s ORF- Finder [52]. Results of ORF-Finder were used to manually curate the gene models observing rules of intron-exon junction and the subsequent sequences re-blasted against NCBI’s non-redundant database to confirm homology before inclusion into the genes list. Genes with incomplete or no conserved functional domains were considered putative pseudogenes.

The identified *Glossina* genes were renamed after their closest *Drosophila* homologs for easier comparison. Abbreviations (Ga–*G*. *austeni*, Gbr-*G*. *brevipalpis*, Gff-*G*. *f fuscipes*, Gmm-*G*. *m*. *morsitans* and Gpd-*G*. *pallidipes*) of the species names were used as prefixes to the specific gene name to identify them. The *G*. *m*. *morsitans* OBPs without homologs in *D*. *melanogaster* were named as described by Liu and colleagues [24].

### Comparative Phylogenetic Analysis of *Glossina* Chemosensory Genes

Multiple sequence alignments for each class of the chemosensory genes were generated using MUSCLE v3.6 [53] with default settings. Resulting alignmentswere manually edited using standalone Jalview v2 [54] (S2 Dataset), then converted into Phylip format using ClustalX v2.1 [55]. The best substitution model for the alignment was determined using ProtTest server v3.2.1 [56]. Phylogeny inference for the aligned sequences were deduced using a Maximum-likelihood approach as implemented in RAxML v8.2.0 [57] with 1000 bootstrap iterations. Obtained phylogenetic trees were viewed and rendered using Fig Tree viewer v1.4.1. Based on their relationship to other species in the tree of life [43], *D*. *melanogaster* and *An*. *gambiae* were used as out groups.

### Selection Analysis

Codon alignment of *Glossina* orthologs was done using Prank v 140603 [58] and their corresponding phylogenetic trees constructed using RAxML v8.2.0 [57]. Signatures of natural selection on orthologs were evaluated by calculating ratios of nonsynonymous to synonymous substitutions (*d*_N_/*d*_S_) in codeml in PAML package v4 [59]. Three site models including M1a (Nearly neutral), M2a (Positive Selection) and M8 (beta & w) were evaluated against their null models to test for selection using log-likelihood ratio (LRT). In case of duplicates, copies of gene loci were separated in order to assess the levels of selection across intra-species paralogs. Corresponding *p*-value was calculated to test for significance of selection. A p-value < = 0.05 was used to consider a gene to be under positive selection. Similarly, selection analysis was carried out using HyPhy package [60] hosted on Datamonkey web server [41]. In this case, neighbor joining trees were constructed within the package and an appropriate model of nucleotide evolution was determined for each alignment, prior to analysis. Two algorithms; Mixed effects model of Evolution (MEME) [61] and PARRIS [62] were used to identify sites under episodic selection taking recombination events into account. A *p*-value < = 0.05 was implemented to estimate the rate of false positives (type I error) in which neutrally evolving sites may be erroneously reported to be under selection.

### Accession Numbers

Accession numbers of *Glossina spp*. annotated chemosensory proteins and those used in comparative analysis. *Glossina* ids were retrieved from Vectrobase alongside those of *Anopheles gambiae* and *Musca domestica*. Uniprot accession ids are provided for *Drosophila melanogaster* while those of *Ceratitis capitata* are from Genebank.

#### Odorant binding proteins

*Glossina austeni*

GAUT003576-PA,GAUT045923-PA,GAUT045912-PA,GAUT045925-PA,GAUT045144-PA,GAUT048147-PA,GAUT018078-PA,GAUT030435-PA,GAUT041055-PA,GAUT039149-PA,GAUT028974-PA,GAUT051622-PA,GAUT040992-PA,GAUT029308-PA,GAUT028968-PA,GAUT026721-PA,GAUT019500-PA,GAUT029664-PA,GAUT019501-PA,GAUT019501-PA,GAUT030010-PA,GAUT030009-PA,GAUT030008-PA,GAUT044447-PA,GAUT043978-PA,GAUT051640-PA,GAUT051645-PA,GAUT051620-PA

*Glossina brevipalpis*

GBRI030526-PA,GBRI036202-PA,GBRI035551-PA,GBRI035552-PA,GBRI035549-PA,GBRI010734-PA,GBRI012886-PA,GBRI045128-PA,GBRI026688-PA,GBRI016471-PA,GBRI016436-PA,GBRI010929-PA,GBRI040269-PA,GBRI036199-PA,GBRI041963-PA,GBRI013864-PA,GBRI031755-PA,GBRI031753-PA,GBRI031754-PA,GBRI031756-PA,GBRI031703-PA,GBRI031705-PA,GBRI031704-PA,GBRI023685-PA,GBRI009351-PA,GBRI012898-PA,GBRI012882-PA

*Glossina fuscipes fuscipes*

GFUI025618-PA,GFUI007906-PA,GFUI000760-PA,GFUI000759-PA,GFUI000757-PA,GFUI048313-PA,GFUI004675-PA,GFUI008988-PA,GFUI008564-PA,GFUI009068-PA,GFUI007894-PA,GFUI026749-PA,GFUI040667-PA,GFUI048612-PA,GFUI048613-PA,GFUI048614-PA,GFUI049167-PA,GFUI004156-PA,GFUI004155-PA,GFUI027466-PA,GFUI045274-PA,GFUI035804-PA,GFUI035776-PA

*Glossina morsitans morsitans*

GMOY008038-PA,GMOY009475-PA,GMOY001927-PA,GMOY005386-PA,GMOY004772-PA,GMOY010761-PA,GMOY001365-PA,GMOY003305-PA,GMOY009271-PA,GMOY006265-PA,GMOY011399-PA,GMOY006479-PA,GMOY006480-PA,GMOY005796-PA,GMOY005084-PA,GMOY010839-PA,GMOY003312-PA,GMOY004392-PA,GMOY007472-PA,GMOY005479-PA,GMOY012018-RB,GMOY012323-PA,GMOY012193-PA,GMOY012195-PA,GMOY012218-PA,GMOY012239-PA,GMOY012253-PA,GMOY012276-PA,GMOY012356-PA,GMOY012357-PA,GMOY005610-PA

*Glossina pallidipes*

GPAI017685-PA,GPAI006440-PA,GPAI032191-PA,GPAI032193-PA,GPAI032197-PA,GPAI018668-PA,GPAI045033-PA,GPAI017770-PA,GPAI004501-PA,GPAI008752-PA,GPAI008777-PA,GPAI018009-PA,GPAI008860-PA,GPAI009631-PA,GPAI013560-PA,GPAI013557-PA,GPAI013558-PA,GPAI013555-PA,GPAI031702-PA,GPAI031704-PA,GPAI031703-PA,GPAI005408-PA,GPAI041909-PA,GPAI045017-PA,GPAI045022-PA,GPAI045024-PA

*D. melanogaster*

O02372,Q27377,P54192,Q9V8Y9,Q9V8Y2,Q8MMF9,P54193,Q9VAJ4,Q9VAI6,Q8SY61,Q9V931,Q23970,Q9VR94,P54195,Q8MKJ4,Q9V938,P54194,P54191,P54185,Q8MKK0,A1ZBQ4,Q9W372,Q9VR95,Q9VNL2,Q7JVM1,Q9VAI7,Q7KE33,Q9VWM0,Q7KE32,Q7K084,Q9VR96,D1FYT3,Q4V3N1,Q7K088,Q8MVX6,D1FYH5,Q9VNL1,A1Z8I9,A1Z8E4,A1ZBP9,Q9VHQ9,A1ZBQ3,A1ZBP7,Q9W209,A1Z9Q5,A1Z9Q6,Q7KUQ3,Q86BF9,A1Z9Q2,Q9VDE1,A1Z8E3,A1Z9Q4,Q8T6R8,E2DBU7,E2DCD5,A9QK61

*M. domestica*

MDOA007276-PA,MDOA004728-PA,MDOA013142-PA,MDOA009850-PA,MDOA014153-PA,MDOA012315-PB,MDOA007587-PA,MDOA000539-PA,MDOA009520-PA,MDOA000889-PA,MDOA012315-PA,MDOA010320-PA,MDOA006902-PA,MDOA005410-PA,MDOA012373-PA,MDOA013526-PA,MDOA013340-PA,MDOA011898-PA,MDOA012293-PA,MDOA005617-PA,MDOA014993-PA,MDOA002810-PA,MDOA009465-PA,MDOA003634-PA,MDOA011594-PA,MDOA004718-PA,MDOA005255-PA,MDOA012958-PA,MDOA012814-PA,MDOA008946-PA,MDOA008603-PA,MDOA008774-PA,MDOA003332-PA,MDOA003832-PA,MDOA001753-PA,MDOA003303-PA,MDOA010146-PA,MDOA011279-PA,MDOA015523-PA,MDOA011147-PA,MDOA011314-PA,MDOA003913-PA,MDOA012317-PA,MDOA005286-PA,MDOA013466-PA,MDOA003735-PA,MDOA012772-PA,MDOA008740-PA,MDOA000714-PA,MDOA002286-PA,MDOA013644-PA,MDOA000734-PA,MDOA002802-PA,MDOA009637-PA,MDOA013698-PA,MDOA004040-PA,MDOA007337-PA,MDOA010340-PA,MDOA001064-PA,MDOA003787-PA,MDOA014452-PA,MDOA010806-PA,MDOA004094-PA,MDOA004456-PA,MDOA008804-PA,MDOA003429-PA,MDOA003694-PA,MDOA004433-PA,MDOA001399-PA,MDOA002259-PA,MDOA009815-PA,MDOA004406-PA,MDOA003400-PA,MDOA004070-PA,MDOA014188-PD,MDOA014188-PG,MDOA004116-PA,MDOA001908-PA

*Ceratitis capitata*

XM_004521128.1,XM_004524969.1,XM_004524970.1,XM_004524978.1,XM_00425083.1,XM_004254959.1,XM_004517746.1,XM_004518409.1,XM_004523388.1,XM_004523387.1,XM_004529312.1,XM_0045211129.1,XM_004521127.1

#### Chemosensory receptors

*Glossina austeni*

GAUT014421-PA, GAUT038415-PA,GAUT027332-PA,GAUT027343-PA,GAUT046063-PA

*Glossina brevipalpis*

GBRI045129-PA, GBRI011414-PA,GBRI020682-PA,GBRI020713-PA

*Glossina fuscipes fuscipes*

GFUI014924-PA, GFUI040903-PA,GFUI003186-PA,GFUI003196-PA,GFUI039843-PA

*Glossina morsitans morsitans*

GMOY010026-PA, GMOY010882-PA,GMOY012164-PA,GMOY010874-PA,GMOY009731-PA

*Glossina pallidipes*

GPAI012674-PA, GPAI011776-PA,GPAI029774-PA,GPAI029784-PA,GPAI031814-PA

*Drosophila melanogaster*

Q8MLP9, Q9W0X2, D5A7M1, Q27377

*Musca domestica*

MDOA006615-PA, MDOA008546-PA, MDOA001428-PA,MDOA000806-PA,MDOA008937-PA

*An.gambaie*

AGAP008058-PA, AGAP008055-PA, AGAP008059-PA,AGAP008062-PA,AGAP008052-PA,AGAP008051-PA,AGAP008054-PA

#### Sensory neuron membrane proteins

*Glossina austeni*

GAUT049266-PA, GAUT008732-PA

*Glossina breviplapis*

GBRI029848-PA, GBRI009197-PA

*Glossina fuscipes fuscipes*

GFUI000887-PA, GFUI009502-PA

*Glossina morsitans morsitans*

GMOY002994-PA, GMOY006180-PA

*D. melanogaster*

Q9VDD3, E1JI63

*M. domestica*

MDOA006272-PB,MDOA006435-PA

*An.gambaie*

AGAP002451-PA,AGAP005716-PAhttp://www.uniprot.org/uniprot/E1JI63

#### Gustatory receptors

*Glossina austeni*

GAUT050702-PA,GAUT041339-PA,GAUT018372-PA,GAUT018371-PA,GAUT037007-PA,GAUT018378-PA,GAUT030746-PA,GAUT018082-PA,GAUT016799-PA,GAUT032734-PA,GAUT042077-PA,GAUT025297-PA,GAUT018813-PA

*Glossina brevipalpis*

GBRI008315-PA,GBRI004163-PA,GBRI016968-PA,GBRI016977-PA,GBRI039848-PA,GBRI043822-PA,GBRI043906-PA,GBRI014933-PA

*Glossina fuscipes fuscipes*

GFUI005702-PA,GFUI034303-PA,GFUI041369-PA,GFUI018032-PA,GFUI027606-PA,GFUI026404-PA,GFUI051944-PA,GFUI022205-PA,GFUI025370-PA,GFUI036605-PA,GFUI041074-PA

*Glossina morsitans morsitans*

GMOY008001-PA,GMOY003231-PA,GMOY004207-PA,GMOY007472-PA,GMOY011615-PA,GMOY006209-PA,GMOY011510-PA,GMOY011903-PA,GMOY005361-PA

*Glossina pallidipes*

GPAI014620-PA,GPAI045887-PA,GPAI035388-PA,GPAI037163-PA,GPAI019874-PA,GPAI039461-PA,GPAI004494-PA,GPAI040289-PA,GPAI040385-PA,GPAI007341-PA,GPAI024994-PA,GPAI040381-PA,GPAI043562-PA

*D. melanogaster*

Q9W497,Q9VSH2,P83293,Q9W367,P58950,P58952,P58953,P58954,Q9V4K2,P58962,P83295,Q9VZJ6,P83297,Q9W0M2,Q9VD76,P83296,Q9VTN0,Q9VYZ2,P84181,Q8IRL8,Q9VJF2,Q9W2B2,P58955,Q8INZ2,Q8IN58,Q8INM9,Q9VEU0,Q9VB26,Q8IMN5,Q8IMN6,Q9VB30,Q0E9G8,H0RNL7,D3PK93,E1JJC5,Q8MLS6,Q7KV53,Q8IN22,M9PAZ2,M9PGM7,A1Z881,M9PBP0,Q9W1V0,B4PH96,B4PH99,B4PHA1,B4PX40,B4PHA0,B4PH98,B4PZC5,B4PH97,B6ZDW0

*M. domestica*

MDOA000140-PA,MDOA000302-PA,MDOA000316-PA,MDOA000580-PA,MDOA000804-PA,MDOA000952-PA,MDOA001249-PA,MDOA002394-PA,MDOA002976-PA,MDOA002995-PA,MDOA003120-PA,MDOA003761-PA,MDOA003814-PA,MDOA004047-PA,MDOA004843-PA,MDOA004883-PA,MDOA005532-PA,MDOA006053-PA,MDOA006078-PA,MDOA006341-PA,MDOA006396-PA,MDOA006542-PA,MDOA007003-PA,MDOA007173-PA,MDOA007349-PA,MDOA007502-PA,MDOA008622-PA,MDOA008716-PA,MDOA008860-PA,MDOA008965-PA,MDOA009078-PA,MDOA009179-PA,MDOA009364-PA,MDOA009614-PA,MDOA009686-PA,MDOA009754-PA,MDOA009880-PA,MDOA011018-PA,MDOA011119-PA,MDOA011281-PA,MDOA012391-PA,MDOA012949-PA,MDOA013669-PA,MDOA014425-PA,MDOA014604-PA,MDOA015305-PA,MDOA002641-PA,MDOA002364-PA,MDOA014947-PA,MDOA015347-PA

*An.gambiae*

AGAP004716-PA,AGAP004727-PA,AGAP005047-PA,AGAP005495-PA,AGAP005514-PA,AGAP006143-RD,AGAP006399-PA,AGAP006450-PA,AGAP006713-RA,AGAP006716-PA,AGAP006717-PA,AGAP006874-PA,AGAP006875-PA,AGAP006876-PA,AGAP006877-RB,AGAP006917-PA,AGAP001915-PA,AGAP002633-PA,AGAP002635-RA,AGAP001125-PA,AGAP003098-PA,AGAP003256-PA,AGAP003255-PA,AGAP003254-PA,AGAP003253-PA,AGAP003260-PA,AGAP003259-RA,AGAP004114-PA,AGAP001171-PA,AGAP001172-PA,AGAP001173-PA,AGAP001170-PA,AGAP001169-RA,AGAP004313-PA,AGAP002275-PA,AGAP011915-PA,AGAP007757-PA,AGAP009256-RA,AGAP009802-PA,AGAP009803-PA,AGAP009804-PA,AGAP009805-RA,AGAP009853-PA,AGAP009854-PA,AGAP009856-PA,AGAP009857-PA,AGAP009858-PA,AGAP009999-PA,AGAP009855-PA,AGAP007756-PA,AGAP012713-PA,AGAP001114-PA,AGAP001117-RA,AGAP001119-PA,AGAP001122-PA,AGAP001123-PA,AGAP001121-PA,AGAP001120-PA,AGAP001115-PA,AGAP001137-PA,AGAP010195-PA,

#### Odorant receptors

*Glossina austeni*

GAUT014395-PA,GAUT050371-PA,GAUT004311-PA,GAUT045920-PA,GAUT028888-PA,GAUT021583-PA,GAUT000836-PA,GAUT050213-PA,GAUT050213-PA,GAUT022268-PA,GAUT044021-PA,GAUT022034-PA,GAUT028238-PA,GAUT011101-PA,GAUT016620-PA,GAUT005608-PA,GAUT042364-PA,GAUT042360-PA,GAUT018044-PA,GAUT003629-PA,GAUT038273-PA,GAUT018383-PA,GAUT032244-PA,GAUT021320-PA,GAUT051820-PA,GAUT021321-PA,GAUT035779-PA,GAUT050214-PA,GAUT003281-PA,GAUT005460-PA,GAUT006649-PA,GAUT040462-PA,GAUT036655-PA,GAUT005363-PA,GAUT034813-PA

*Glossina brevipalpis*

GBRI045111-PA,GBRI018062-PA,GBRI036522-PA,GBRI035583-PA,GBRI036342-PA,GBRI002464-PA,GBRI016989-PA,GBRI044639-PA,GBRI034666-PA,GBRI009897-PA,GBRI026647-PA,GBRI008361-PA,GBRI028428-PA,GBRI026891-PA,GBRI015995-PA,GBRI011898-PA,GBRI011904-PA,GBRI011358-PA,GBRI031244-PA,GBRI031534-PA,GBRI002179-PA,GBRI026158-PA,GBRI017432-PA,GBRI017598-PA,GBRI040021-PA,GBRI044640-PA,GBRI018811-PA,GBRI027004-PA,GBRI041284-PA,GBRI030235-PA,GBRI005734-PA,GBRI013056-PA,GBRI012762-PA,GBRI030714-PA

*Glossina fuscipes fuscipes*

GFUI014938-PA,GFUI043297-PA,GFUI032492-PA,GFUI028755-PA,GFUI007794-PA,GFUI003104-PA,GFUI003105-PA,GFUI003499-PA,GFUI028213-PA,GFUI008162-PA,GFUI032116-PA,GFUI005658-PA,GFUI037305-PA,GFUI034469-PA,GFUI045476-PA,GFUI009257-PA,GFUI038138-PA,GFUI038147-PA,GFUI042981-PA,GFUI027054-PA,GFUI051694-PA,GFUI007388-PA,GFUI043789-PA,GFUI036188-PA,GFUI022534-PA,GFUI022472-PA,GFUI003500-PA,GFUI053522-PA,GFUI022126-PA,GFUI047908-PA,GFUI049134-PA,GFUI037003-PA,GFUI024278-PA,GFUI012941-PA,GFUI035140-PA

*Glossina morsitans morsitans*

GMOY008038-PA,GMOY009475-PA,GMOY001927-PA,GMOY005386-PA,GMOY004772-PA,GMOY010761-PA,GMOY001365-PA,GMOY003305-PA,GMOY009271-PA,GMOY006265-PA,GMOY011399-PA,GMOY006479-PA,GMOY006480-PA,GMOY005796-PA,GMOY005084-PA,GMOY010839-PA,GMOY003312-PA,GMOY004392-PA,GMOY007472-PA,GMOY005479-PA,GMOY012018-RB,GMOY012323-PA,GMOY012193-PA,GMOY012195-PA,GMOY012218-PA,GMOY012239-PA,GMOY012253-PA,GMOY012276-PA,GMOY012356-PA,GMOY012357-PA,GMOY005610-PA

*Glossina pallidipes*

GPAI034871-PA,GPAI027642-PA,GPAI015219-PA,GPAI004010-PA,GPAI034198-PA,GPAI039623-PA,GPAI039631-PA,GPAI031316-PA,GPAI031326-PA,GPAI029610-PA,GPAI041951-PA,GPAI026906-PA,GPAI014680-PA,GPAI009882-PA,GPAI009200-PA,GPAI039539-PA,GPAI001497-PA,GPAI004557-PA,GPAI045424-PA,GPAI045426-PA,GPAI039747-PA,GPAI017649-PA,GPAI041241-PA,GPAI037164-PA,GPAI033169-PA,GPAI012943-PA,GPAI012945-PA,GPAI046202-PA,GPAI002749-PA,GPAI042230-PA,GPAI031315-PA,GPAI024118-PA,GPAI001626-PA,GPAI040919-PA,GPAI002024-PA,GPAI004056-PA,GPAI027550-PA,GPAI009882-PA,GPAI035133-PA

*D. melanogaster*

Q9VPT1,Q9VZL7,P81909,P81910,O46077,Q9V3Q2,P81915,P81917,P81921,Q9VNB5,Q9I816,Q9VXL0,Q9VYZ1,Q9W5G6,P81912,P81911,P81913,Q9VLE5,P81916,P81914,Q9V9I2,Q9V589,P81919,P81922,P81918,Q9V3N2,Q9V9I4,Q9V6A9,Q9V6H2,Q9V568,Q9V8Y7,Q9W1P8,P81923,P82982,Q9VT90,Q9VT92,Q9VT08,Q9VT20,Q9VVF3,Q9W3I5,Q9VHQ7,Q9VHE6,Q9VHS4,Q9VFN2,P82986,Q9VAZ3,Q9W2U9,Q8IRZ5,Q9VZW8,Q9VNB3,Q9VHQ6,Q9VCS9,Q9VCS8,E1JIA4,M9NFD3,E2E626,E2E5L1,E2E5L0,E2E510,B4NY14

*M. domestica*

MDOA000926-PA,MDOA000137-PA,MDOA000385-PA,MDOA000464-PA,MDOA001095-PA,MDOA001330-PA,MDOA001508-PA,MDOA001711-PA,MDOA001967-PA,MDOA002017-PA,MDOA002113-PA,MDOA002222-PA,MDOA002540-PA,MDOA002654-PA,MDOA002736-PA,MDOA002822-PA,MDOA002922-PA,MDOA003091-PA,MDOA003495-PA,MDOA003512-PA,MDOA003540-PA,MDOA003948-PA,MDOA004405-PA,MDOA004757-PA,MDOA004936-PA,MDOA004949-PA,MDOA005313-PA,MDOA005821-PA,MDOA005976-PA,MDOA006361-PA,MDOA006570-PA,MDOA006773-PA,MDOA006970-PA,MDOA007213-PA,MDOA007232-PA,MDOA007549-PA,MDOA007555-PA,MDOA007822-PA,MDOA007881-PA,MDOA008272-PA,MDOA008672-PA,MDOA008787-PA,MDOA009136-PA,MDOA009183-PA,MDOA009203-PA,MDOA009938-PA,MDOA010127-PA,MDOA010179-PA,MDOA010267-PA,MDOA010394-PA,MDOA010396-PA,MDOA010576-PA,MDOA011183-PA,MDOA011663-PA,MDOA011814-PA,MDOA011954-PA,MDOA012084-PA,MDOA012436-PA,MDOA012443-PA,MDOA012722-PA,MDOA012767-PA,MDOA012864-PA,MDOA012897-PA,MDOA012955-PA,MDOA013188-PA,MDOA013204-PA,MDOA013213-PA,MDOA013229-PA,MDOA013697-PA,MDOA014353-PA,MDOA014482-PA,MDOA014540-PA,MDOA014647-PA,MDOA014744-PA,MDOA014843-PA,MDOA014864-PA,MDOA014904-PA,MDOA015346-PA,MDOA015469-PA,MDOA015496-PA,MDOA015498-PA,MDOA005448-PA,MDOA007080-PA,MDOA007097-PA,MDOA010057-PA,MDOA013717-PA

#### Ionotropic & ionotropic glutatmate receptors

*Glossina austeni*

GAUT036857-PA,GAUT010844-PA,GAUT032862-PA,GAUT032862-PA,GAUT018821-PA,GAUT036856-PA,GAUT051652-PA,GAUT029664-PA,GAUT011688-PA,GAUT019628-PA,GAUT028361-PA,GAUT017831-PA,GAUT035430-PA,GAUT051179-PA,GAUT013397-PA,GAUT013397-PA,GAUT003875-PA,GAUT051343-PA,GAUT037856-PA,GAUT038749-PA,GAUT002274-PA,GAUT026102-PA,GAUT023024-PA,GAUT005991-PA,GAUT026111-PA,GAUT031582-PA,GAUT008471-PA,GAUT032864-PA

*Glossina brevipalpis*

GBRI004368-PA,GBRI037007-PA,GBRI006509-PA,GBRI004366-PA,GBRI004366-PA,GBRI013356-PA,GBRI037007-PA,GBRI012928-PA,GBRI001929-PA,GBRI023337-PA,GBRI000712-PA,GBRI039411-PA,GBRI033584-PA,GBRI012051-PA,GBRI033291-PA,GBRI016181-PA,GBRI016181-PA,GBRI012020-PA,GBRI018928-PA,GBRI009997-PA,GBRI002787-PA,GBRI010267-PA,GBRI006799-PA,GBRI006802-PA,GBRI006799-PA,GBRI029815-PA,GBRI013857-PA,GBRI040612-PA

*Glossina fuscipes fuscipes*

GFUI019198-PA,GFUI016186-PA,GFUI018591-PA,GFUI019200-PA,GFUI019200-PA,GFUI031610-PA,GFUI041857-PA,GFUI035802-PA,GFUI017944-PA,GFUI008852-PA,GFUI031962-PA,GFUI025996-PA,GFUI041337-PA,GFUI028023-PA,GFUI019558-PA,GFUI029180-PA,GFUI029178-PA,GFUI043801-PA,GFUI005590-PA,GFUI004860-PA,GFUI020203-PA,GFUI000065-PA,GFUI009601-PA,GFUI000460-PA,GFUI000063-PA,GFUI045184-PA,GFUI050910-PA

*Glossina morsitans morsitans*

GMOY004222,GMOY012186,GMOY006490,GMOY007988,GMOY001514,GMOY012037,GMOY006751,GMOY001810,GMOY004959,GMOY005753,GMOY007825,GMOY000804,GMOY012048,GMOY012127,GMOY008789,GMOY008540,GMOY012136,GMOY006890,GMOY009209,GMOY002585,GMOY004997,GMOY009750,GMOY004578

*Glossina pallidipes*

GPAI011564-PA,GPAI006854-PA,GPAI010111-PA,GPAI011561-PA,GPAI011561-PA,GPAI019869-PA,GPAI006854-PA,GPAI045043-PA,GPAI016226-PA,GPAI011331-PA,GPAI007758-PA,GPAI004624-PA,GPAI022505-PA,GPAI032358-PA,GPAI017485-PA,GPAI036018-PA,GPAI036018-PA,GPAI025294-PA,GPAI027894-PA,GPAI044391-PA,GPAI022870-PA,GPAI042411-PA,GPAI006139-PA,GPAI006142-PA,GPAI029067-PA,GPAI010422-PA,GPAI006139-PA,GPAI006944-PA

*D. melanogaster*

Q9W365,Q9W3P2,A1Z882,E9NA96,A1Z8N9,B7Z069,Q9VCM4,A1ZBM8,M9PCT4,A1ZBM7,Q2MGM0,B7YZQ4,B7Z0P2,A1Z8P2,Q9VCM0,Q9W191,Q9VDH6,Q9VYN4,Q9VVU7,Q9V9T2,Q8IN10,A1ZA17,Q8IN09,B7Z0Y1,A8JNV9,Q9W155,Q9VTH3,B7Z0X5,Q9VDN3,A8JUR3,A1ZBM9,B7Z0X6,Q9VTT6,A1Z6D6,Q9VVL1,Q8IMY8,A1ZAY9,Q8IN08,X2JCB2,A1ZA14,Q9W3P0,Q9W3P4,Q9VRL4,A1ZA16,A1ZBG7,Q9VPI2,Q9V9N1,Q9VHL4,Q8IPB8,Q9VVL2,Q9VCM1,Q9VRI8,A1ZA15,A1Z9Y5,M9PGG3,Q9VFV0,Q8IQE2,B7YZQ6,Q9VIA5,Q9VT09,E9NA95,E9NA98,E9NA99,E7E521

*M. domestica*

MDOA007071-PA,MDOA010874-PA,MDOA004663-PA,MDOA002700-PA,MDOA000608-PA,MDOA010444-PA,MDOA014373-PA,MDOA014396-PA,MDOA009431-PA,MDOA011131-PA,MDOA003227-PA,MDOA000640-PA,MDOA003336-PA,MDOA015494-PA,MDOA004232-PA,MDOA015201-PA,MDOA010345-PA,MDOA012059-PA,MDOA009027-PA,MDOA008354-PA,MDOA001109-PA,MDOA006236-PA,MDOA009699-PA,MDOA007005-PA,MDOA002252-PA,MDOA001895-PA,MDOA012195-PA,MDOA013121-PA,MDOA007819-PA,MDOA012117-PA,MDOA008579-PA,MDOA010627-PA,MDOA002571-PA,MDOA012119-PA,MDOA005930-PA,MDOA008763-PA,MDOA003307-PA,MDOA011360-PA,MDOA009489-PA,MDOA000887-PA,MDOA005477-PA,MDOA003828-PA,MDOA007088-PA,MDOA011259-PA,MDOA015382-PA,MDOA009859-PA,MDOA008038-PA,MDOA008826-PA,MDOA008185-PA,MDOA014666-PA,MDOA011062-PA,MDOA000255-PA,MDOA010951-PA,MDOA012239-PA,MDOA013187-PA,MDOA005137-PA,MDOA007608-PA,MDOA010321-PA,MDOA000493-PA,MDOA003357-PA,MDOA008271-PA,MDOA010652-PA,MDOA004469-PA,MDOA012545-PA,MDOA007828-PA,MDOA005225-PA,MDOA008360-PA,MDOA012330-PA,MDOA011161-PA,MDOA014865-PA,MDOA014404-PA,MDOA008618-PA,MDOA005214-PA,MDOA002045-PA,MDOA006466-PA,MDOA005355-PA,MDOA007990-PA,MDOA012546-PA,MDOA000971-PA,MDOA005099-PA,MDOA005808-PA,MDOA003734-PA,MDOA009668-PA,MDOA006290-PA,MDOA012758-PA,MDOA006255-PA,MDOA002539-PA,MDOA002539-PB,MDOA014635-PA,MDOA004067-PA,MDOA003912-PA,MDOA005542-PA,MDOA004606-PA,MDOA013782-PA,MDOA011463-PA,MDOA011711-PA,MDOA004225-PA,MDOA013355-PA,MDOA000458-PA,MDOA003685-PA,MDOA007697-PA,MDOA001982-PA,MDOA008624-PA,MDOA001178-PA,MDOA009650-PA,MDOA011682-PA,MDOA002092-PA,MDOA002232-PA,MDOA001533-PA,MDOA013906-PA,MDOA007071-PA,

*An.gambiae*

AGAP004923-PA,AGAP004969-PA,AGAP005466-RA,AGAP005527-PA,AGAP005677-PA,AGAP005678-PA,AGAP005679-PA,AGAP006407-PA,AGAP006440-PA,AGAP006691-PA,AGAP007498-PA,AGAP001811-PA,AGAP001812-PA,AGAP013085-PA,AGAP013436-PA,AGAP013242-PA,AGAP013363-PA,AGAP013285-PA,AGAP002763-PA,AGAP013416-PA,AGAP002797-RB,AGAP002904-PA,AGAP013473-PA,AGAP003531-PA,AGAP012951-PA,AGAP013425-PA,AGAP004021-PA,AGAP001478-PA,AGAP004432-PA,AGAP012969-PA,AGAP004475-PA,AGAP013520-PA,AGAP013172-PA,AGAP013409-PA,AGAP000714-PA,AGAP013154-PA,AGAP000803-PA,AGAP000801-RB,AGAP000798-PA,AGAP000140-PA,AGAP000256-PA,AGAP000293-PA,AGAP010411-PA,AGAP011943-PA,AGAP011968-PA,AGAP007951-PA,AGAP008511-PA,AGAP008759-PA,AGAP009014-PA,AGAP010272-PA,AGAP012429-PA,AGAP012447-PA

## Results

### Annotation and Genomic Arrangement of *Glossina* Chemosensory Genes

The numbers of chemosensory gene families identified and annotated in this study are summarized in Table 1 and their metadata in S1 Dataset.

**Table 1:** Summary of putative chemosensory genes annotated in *Glossina* species. *G*. *austeni*, *G*. *brevipalpis*, *G*. *f*. *fuscipes*, *G*. *m*. *morsitans and G*. *pallidipes* against those of selected dipterans.

Species	CSPs^†^	GRs	IRs/IGluRs	OBPs	ORs	SNMPs	Reference(s)
*G*. *austeni*	5	14	28	29	40 (5)	2	**This study**
*G*. *brevipalpis*	4	11	28	28	42 (5)	2	**This study**
*G*. *f*. *fuscipes*	5	14	31 (2)	30 (3)	42 (6)	2	**This study**
*G*. *pallidipes*	5	14	30 (1)	30 (2)	42 (3)	2	**This study**
*G*. *m*. *morsitans*	5	14	30 (2)	30 (3)	46 (3)	2	24,25,31
*An*. *gambiae*	8	76	48	82	79	2	34,68
*D*. *melanogaster*	4	60 (13)	66(9)	52	62 (2)	2	22,64
*M*. *domestica*	5	103	110	>87	86	2	47

^**†**^ CSPs—chemosensory specific proteins, GRs—gustatory receptors, IRs/IGluRs- ionotropic receptors/ionotropic glutamate receptors, OBPs- odorant binding proteins, ORs- odorant receptors, SNMPs- sensory neuron membrane proteins.

Number of genes in parentheses represents putative pseudogenes i.e. either incomplete genes or genes missing functional domain.

Overall, the results presented in Table 1 show that the five tsetse species have fewer chemosensory genes compared to the other dipterans used in this study. Majority of the chemosensory proteins identified in this study (S1 Dataset) contained their respective definitive domains (7tm_7 superfamily in GRs, 7tm_6 in ORs, PBP, ANF- receptor and Lig_Chan in IRs, PBP-GOBP in OBPs, OS-D in CSPs and CD36 in SNMPs). However, a few genes were missing the domain signatures. These included Obp73a in all tsetse species, Obp56h in *G*. *austeni*, Obp20, Or85e and Gr33a in *G*. *brevipalpis*, SNMP1 and Or56a in *G*. *f*. *fuscipes*, and Or67d3 in *G*. *pallidipes*. The GRs and ORs in *G*. *austeni*, *G*. *brevipalpis*, *G*. *f*. *fuscipes*, and *G*. *pallidipes* were 269–480 aa and 295–508 aa long, respectively. Similarly, CSPs and OBPs were 108–178 aa and 108–257 aa long, respectively. The SNMPs and IRs had longer sequences than other gene families, being 384–540 aa and 407–1070 aa long, respectively.

Our analysis revealed a general genome-wide dispersion of the chemosensory genes in all the tsetse species analyzed (S1 Dataset). Fourteen loci were duplicated. The loci included one CSP (Ejbp3; that have two copies namely Ejbp3A and Ejbp3B), three GRs (Gr21a; with three copies namely Gr2a1, Gr2a2 and Gr21a3, Gr28b; two to three copies per genome Gr28bB, Gr28bC, and/or Gr28bD and Gr59f; with two copies: Gr59f1-2). Two OBPs (Obp83a which has four copies; Obp83a1-4, Obp56e; with two copies Obp56e1 and Obp56e2, and eight ORs (Or7a with three copies: Or7a1-3, Or45a with three copies: Or45a1-3, Or67d with five copies: Or67d1-5 and Or56a with two copies: Or56a1 and Or56a2, Or43a, Or46a, Or63a, and Or67c with two copies each). All four copies of Obp83a homolog were in tandem in all the five tsetse genomes, and represented evidence of structural gene variation and rearrangement (S1 Fig, panel A). One of the Obp83a copies was located on the reverse strand. In contrast, duplicated ORs including three copies of Or45a, two copies of Or7a and four to six copies of Or67d homologs were located in different scaffolds (S1 Dataset).

### Comparative Analyses of *Glossina* Chemosensory Gene Families

Sequence alignment of Obp56i and Obp19 from selected dipterans showed variation of amino acids between the third and fourth conserved cysteine residues (labeled C3 and C4 in S2 Fig). *Glossina* Obp56i and Obp19 showed sequence deletions between C3 and C4. In contrast, their homologs from *D*. *melanogaster* and *M*. *domestica* showed amino acid conservation around the same regions.

**Fig 1:**
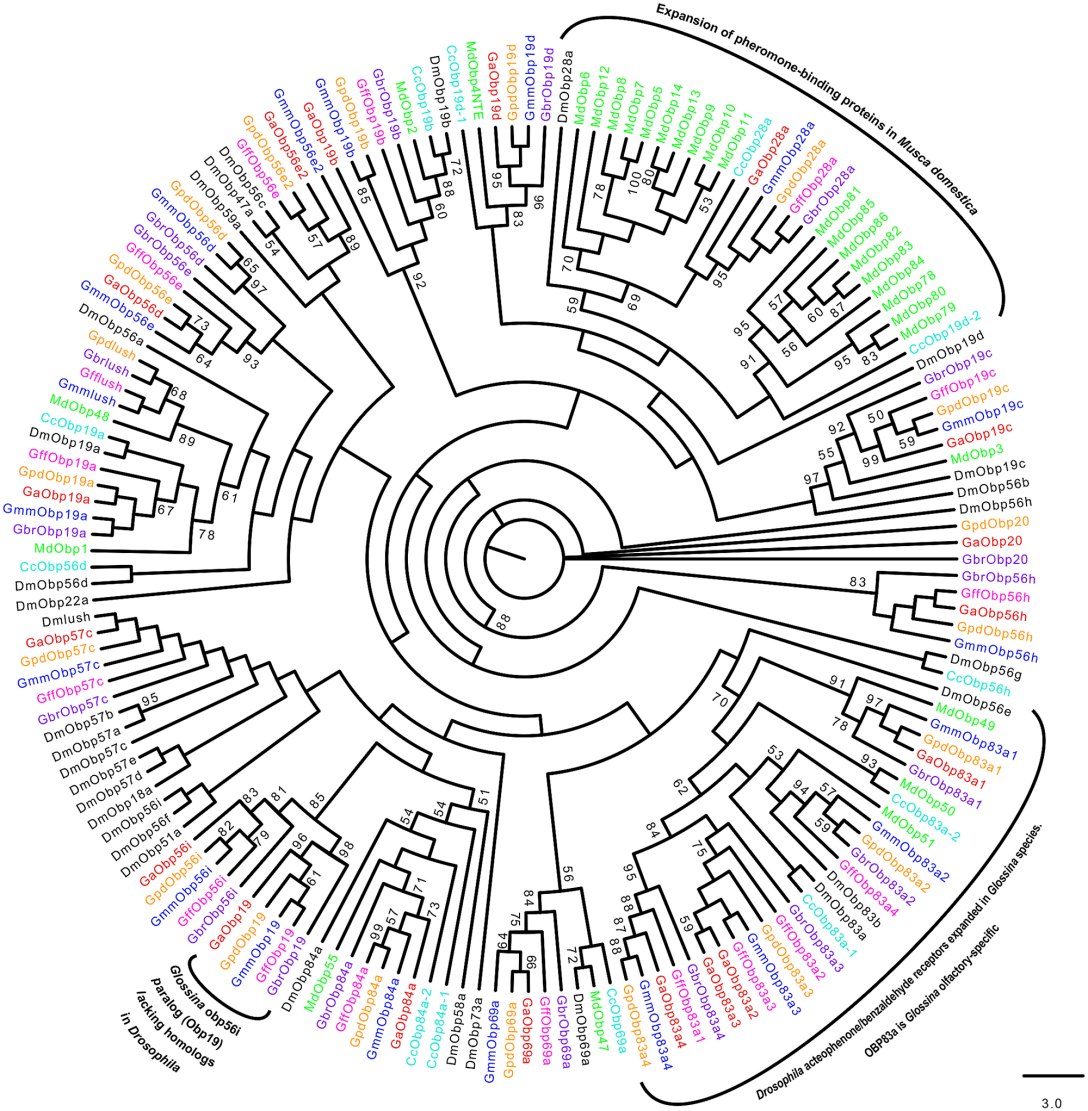
Phylogeny of classic odorant binding proteins. Insect classic OBPs are characterized by six conserved cysteine residues. Different symbols depict OBPs from the different species at the terminal nodes: *Glossina austeni* (red*), *Glossina brevipalpis* (purple*), *Glossina fuscipes fuscipes* (pink*), *Glossina morsitans morsitans* (dark blue*), *Glossina pallidipes* (light orange*), Drosophila melanogaster (black*), *Ceratitis capitata* (sky blue*) *and Musca domestica* (lime green*). The symbol * represents the name of the specific OBP. Sequence alignment was performed using MuSCLE v3.8.31 and phylogeny relationship was inferred using RAxML v8 with best fitting Wheelan and Goldman (WAG) model and 1000 bootstrap iterations.

**Fig 2:**
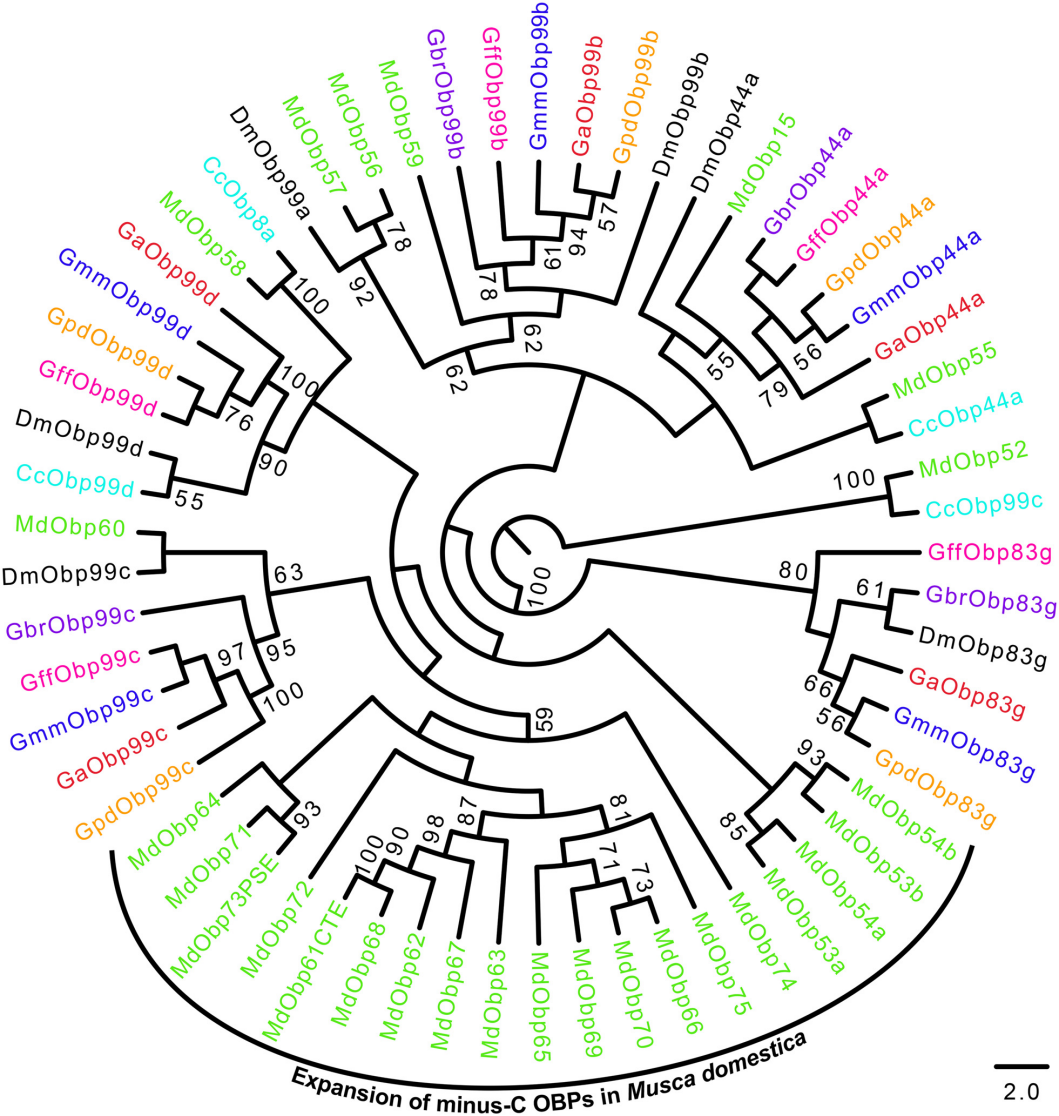
Phylogeny of Minus-C odorant binding proteins. The minus-C OBPs have less than six conserved cysteine residues (Missing C1or C2 and/or C5). Different symbols depict OBPs from the different species at the terminal nodes: *Glossina austeni* (red*), *Glossina brevipalpis* (purple*), *Glossina fuscipes fuscipes* (pink*), *Glossina morsitans morsitans* (dark blue*), *Glossina pallidipes* (light orange*), Drosophila melanogaster (Dm*), *Ceratitis capitata* (sky blue*) *and Musca domestica* (lime green*). The symbol * represents the name of the specific OBP. Sequence alignment was performed using MuSCLE v3.8.31 and phylogeny relationship was inferred using RAxML v8 with best fitting Wheelan and Goldman (WAG) model and 1000 bootstrap iterations.

**Fig 3:**
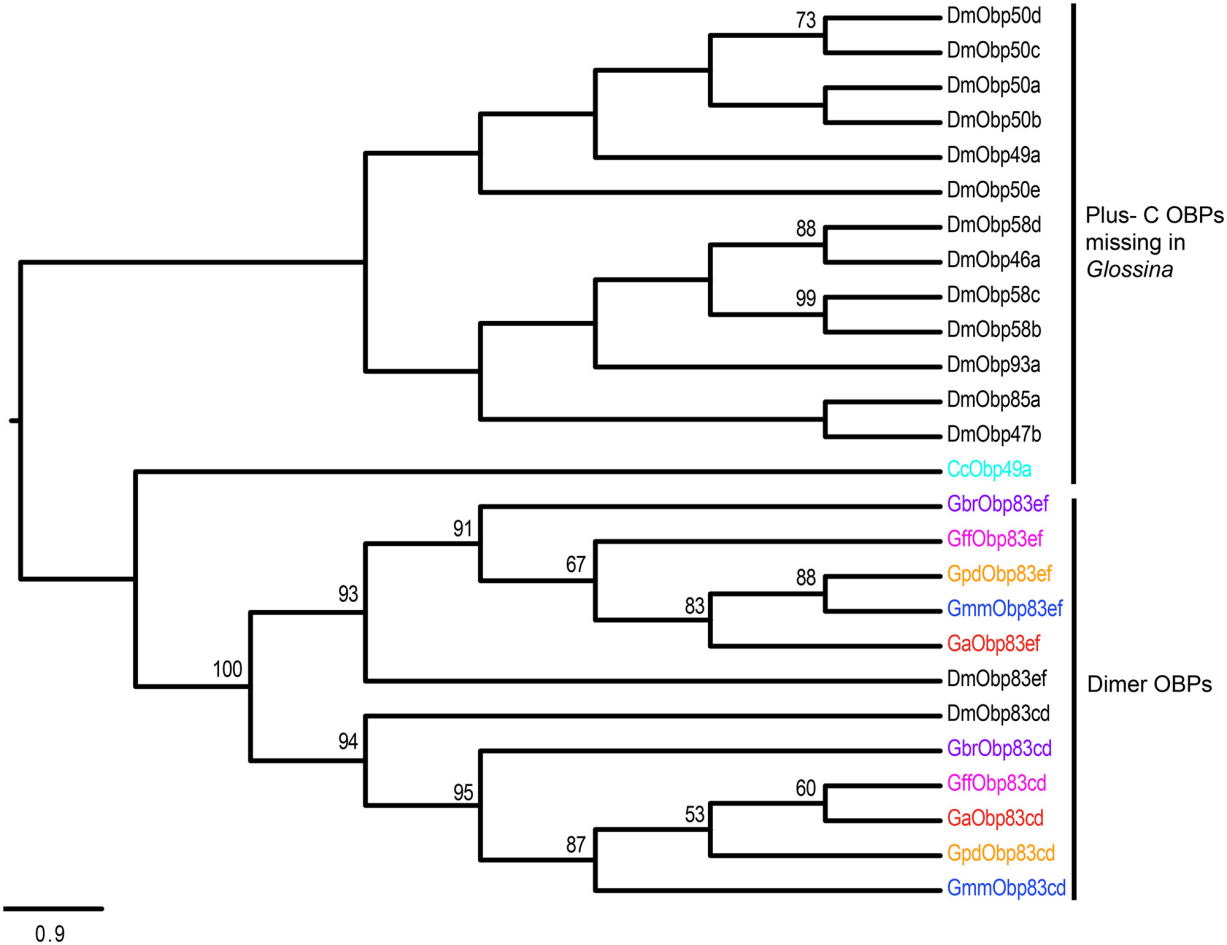
Phylogeny of Plus-C and Classic-Dimer odorant binding proteins. The Plus-C OBPs are characterized by having more than six cysteines and a conserved proline residue. The Classic-dimers have two conserved domains of classic sub-family. Different symbols depict OBPs from the different species at the terminal nodes: *Glossina austeni* (red*), *Glossina brevipalpis* (purple*), *Glossina fuscipes fuscipes* (pink*), *Glossina morsitans morsitans* (dark blue*), *Glossina pallidipes* (light orange*), Drosophila melanogaster (black*), *Ceratitis capitata* (sky blue*) *and Musca domestica* (lime green*). The symbol * represents the name of the specific OBP. Sequence alignment was performed using MuSCLE v3.8.31 and phylogeny relationship was inferred using RAxML v8 with best fitting Wheelan and Goldman (WAG) model and 1000 bootstrap iterations.

**Fig 4:**
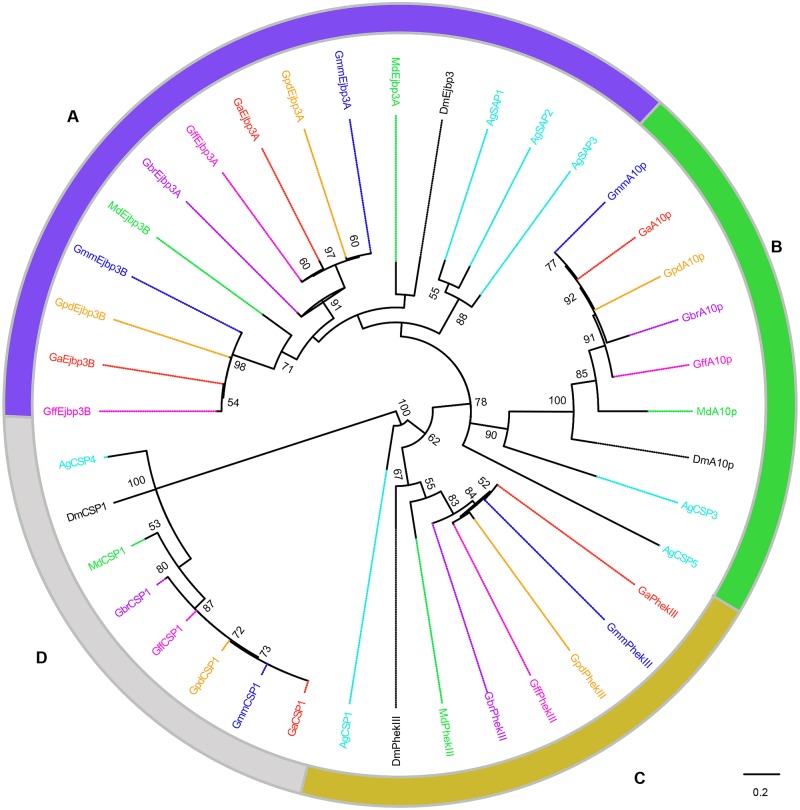
Phylogeny of chemosensory proteins. Clade A shows duplication of ejaculatory bulb protein 3 (Ejbp3 in four tsetse species). Clade B shows expansion of A10p—like homologs in *An*. *gambiae* while clades C and D depicts conservation of Pherokine-3 and CSP1 across the species compared, respectively. Different symbols depict CSPs from the different species at the terminal nodes: *Glossina austeni* (red*), *Glossina brevipalpis* (purple*), *Glossina fuscipes fuscipes* (pink*), *Glossina morsitans morsitans* (dark blue*), *Glossina pallidipes* (light orange*), Drosophila melanogaster (black*), *Anopheles gambiae* (sky blue*) *and Musca domestica* (lime green*). The symbol * represents the name of the specific CSP. Sequence alignment was performed using MuSCLE v3.8.31 and phylogeny relationship was inferred using RAxML v8 with best fitting Wheelan and Goldman (WAG) model and 1000 bootstrap iterations.

**Fig 5:**
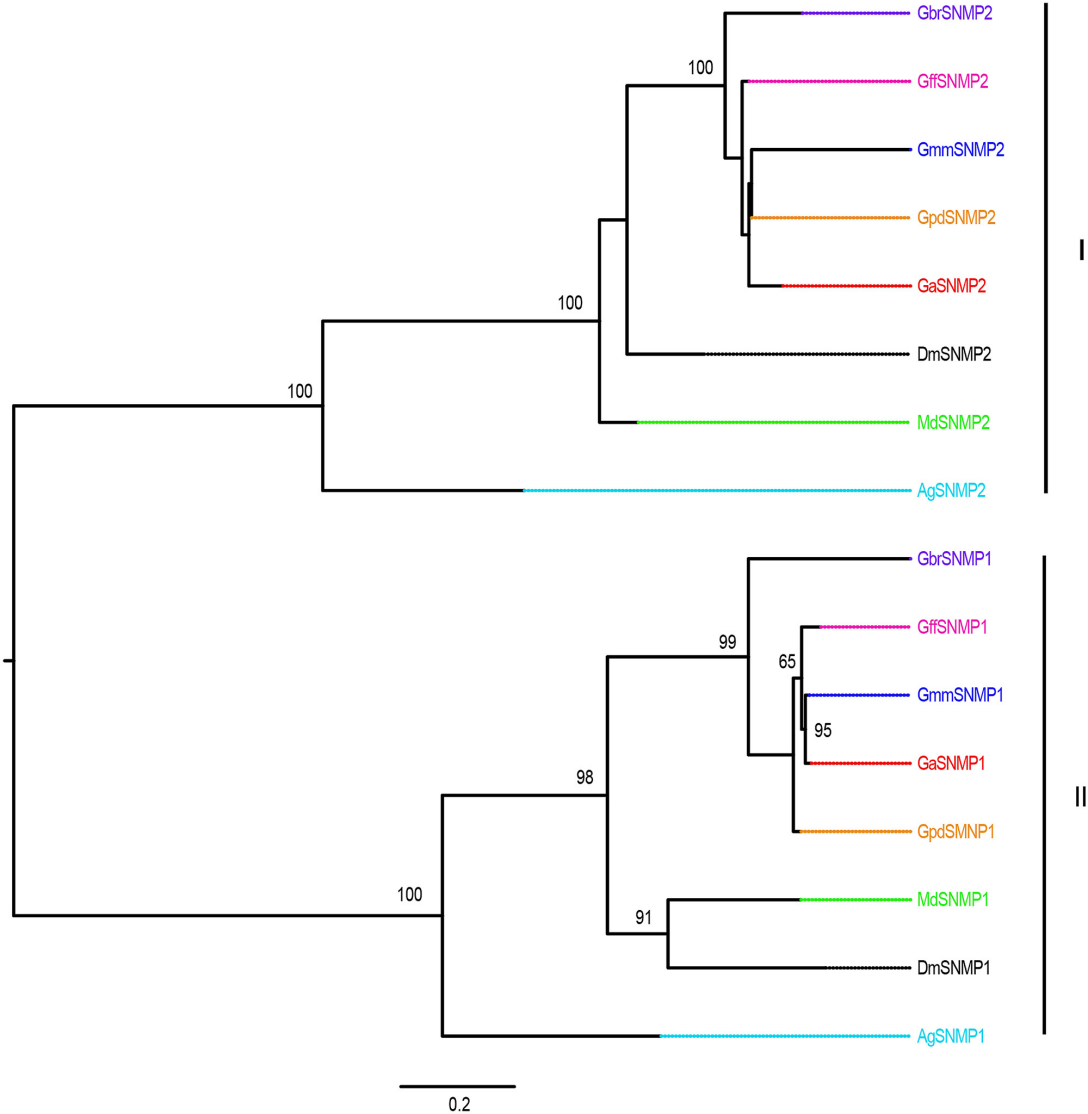
Phylogeny of sensory neuron membrane proteins. Both clades I and II show one to one orthology of the specific SNMP from different insect species. Different symbols depict SNMPs from the different species at the terminal nodes: *Glossina austeni* (red*), *Glossina brevipalpis* (purple*), *Glossina fuscipes fuscipes* (pink*), *Glossina morsitans morsitans* (dark blue*), *Glossina pallidipes* (light orange*), Drosophila melanogaster (black*), *Anopheles gambiae* (sky blue*) *and Musca domestica* (lime green*). The symbol * represents the name of the specific SNMP. Sequence alignment was performed using MuSCLE v3.8.31 and phylogeny relationship was inferred using RAxML v8 with best fitting Wheelan and Goldman (WAG) model and 1000 bootstrap iterations. Phylogenetic relationships of GRs identified in Glossina genes and their homologs in *An*. *gambiae*, *D*. *melanogaster* and *M*. *domestica* are shown in Fig 6. In all the tsetse species, there was expansion of Gr21a, associated with CO_2_ detection in fruit fly and mosquitoes [64,65]. Similarly, expansion of CO2 receptors was noted in *An*. *gambiae* which has expanded Gr63a, a protein co-expressed with Gr21a and involved in CO_2_ detection [65]. No homologs to sugar receptors in *D*. *melanogaster* [66] were identified in any of the five *Glossina species (*Fig 6*)*. Similarly, *D*. *melanogaster* Gr43a, implicated in internal fructose sensing [67] was absent in all tsetse species.

Multiple alignments of the OBPs and CSPs revealed high conservation of conserved cysteine residues (for formation of disulphide bridges) and hydrophobic amino acid residues (for formation ligand-binding sites) (See S2 Dataset). Phylogenetic relationships predicted among the OBPs and CSPs identified in *Glossina* species against those in *C*. *capitata*, *D*. *melanogaster* and *M*. *domestica* are shown in Figs 1–4. About 68.9% (*n* = 29) of the *Glossina* OBPs were grouped into the Classic subfamily (Fig 1) (with six conserved cysteines) while six OBPs in each of the tsetse species were identified into the Minus-C subfamily (with less than the conventional six cysteines) (Fig 2). We did not identify any Plus-C /Atypical subfamily members in any of the *Glossina* species studied (Fig 3). Expansions of Obp56e (two copies) and Obp83a (four copies) classic subfamily were observed in all tsetse species (Fig 1), while *M*. *domestica* and *C*. *capitata* had three and two copies of gene encoding Obp83a respectively. The Obp28a, and Obp19d, were among the list of OBP genes highly expanded in *M*. *domestica* (Fig 1). There were four distinct clades (A–D), of the CSPs (Fig 4). All tsetse species except *G*. *brevipalpis* had two copies of ejaculatory–bulb specific protein 3 (Ejbp3). *G*. *brevipalpis* on the other hand, had a single copy of Ejbp3 similar to *M*. *domestica* (Clade A, Fig 4). Further, orthologs of SNMP1 and SNMP2 reported in *D*. *melanogaster*, *Ae*. *aegypti* and various Lepidoptera species [63] were present in all tsetse species. Two SNMP sub-clades with one-to-one orthology across all insects were identified (Fig 5).

**Fig 6:**
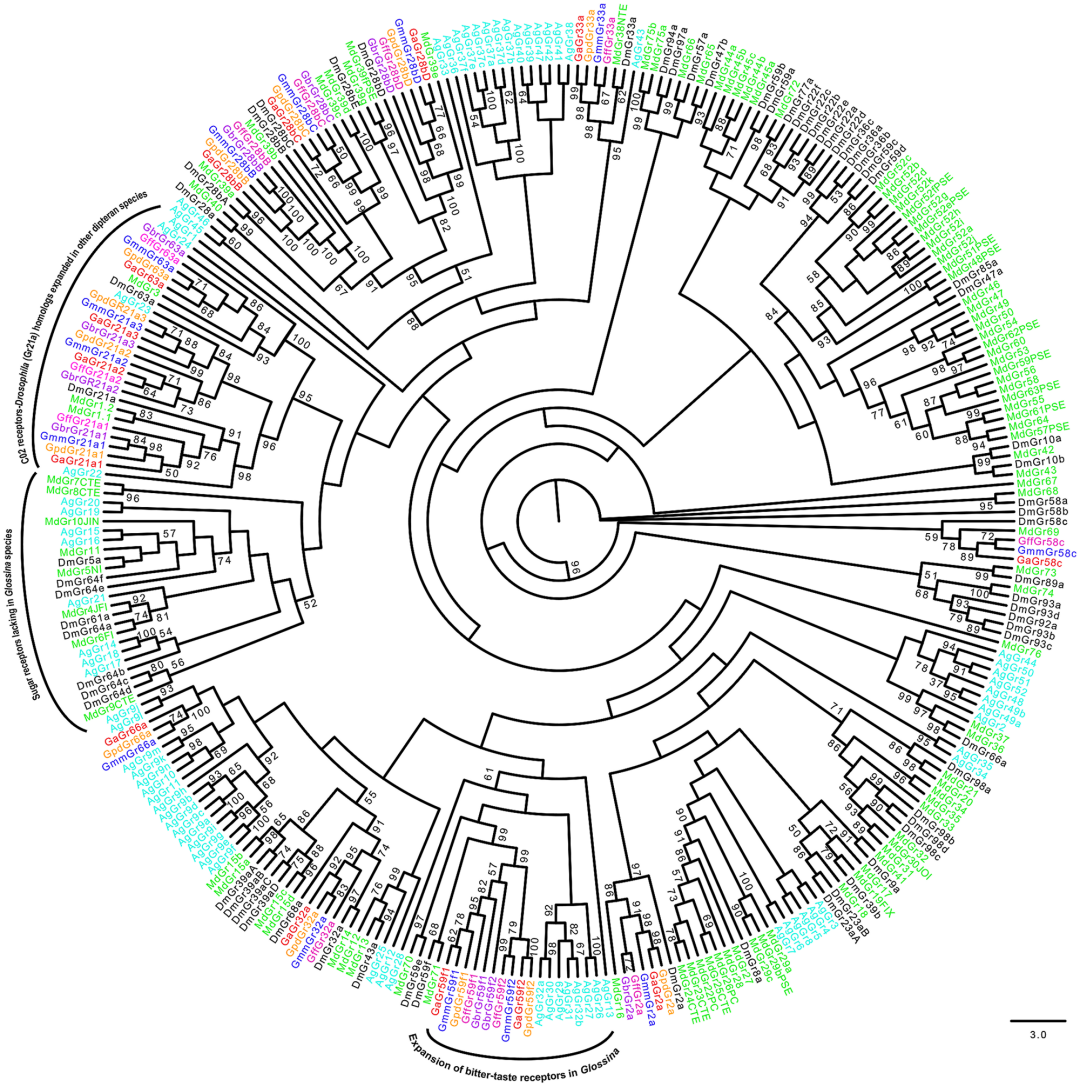
Phylogeny of gustatory receptors. Gustatory receptors responsible for CO_2_ detection show expansion in Glossina species and Musca domestica relative to Drosophila. On the contrary, all receptors responsible for sugar detection are found to be absent in Glossina. Different symbols depict GRs from the different species at the terminal nodes: *Glossina austeni* (red*), *Glossina brevipalpis* (purple*), *Glossina fuscipes fuscipes* (pink*), *Glossina morsitans morsitans* (dark blue*), *Glossina pallidipes* (light orange*), Drosophila melanogaster (black*), *Anopheles gambiae* (sky blue*) *and Musca domestica* (lime green*). The symbol * represents the name of the specific GR. Sequence alignment was performed using MuSCLE v3.8.31 and phylogeny relationship was inferred using RAxML v8 with best fitting Wheelan and Goldman (WAG) model and 1000 bootstrap iterations.

**Fig 7:**
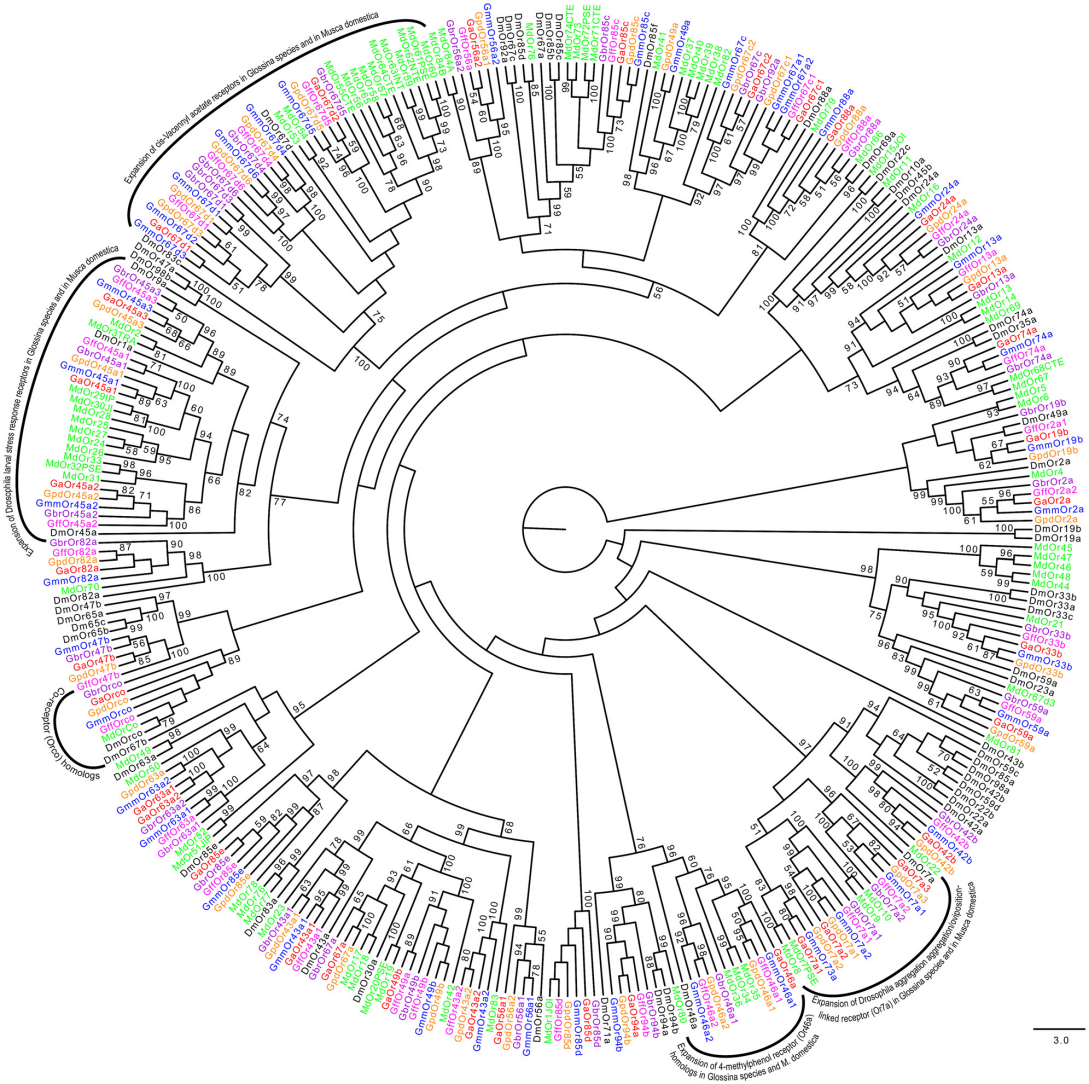
Phylogeny of odorant receptors. Expansion of cis-Vaccenyl acetate receptor (Or67d), 4-Methylphenol receptor (Or46a) and aggregation-linked receptor (Or7a) is observed in *Glossina* species and *Musca domestica* relative to *Drosophila*. Sequence alignment was performed using MuSCLE v3.8.31 and phylogeny relationship was inferred using RAxML v8 with best fitting Wheelan and Goldman (WAG) model and 1000 bootstrap iterations. Different symbols and colors were used to depict ORs from the different species at the terminal nodes: *Glossina austeni* (red*), *Glossina brevipalpis* (purple*), Glossina *fuscipes fuscipes* (pink*), *Glossina morsitans morsit*ans (dark blue*), *Glossina pallidipes* (light orange*), *Drosophila melanogaster* (black*) and *Musca domestica* (lime green*). The symbol * represents the name of the specific OR.

A single copy of the co-receptor (Orco) ortholog was identified in all tsetse species (Fig 7). There were 75–85% amino acid identity between Orco in all tsetse and those of its homologs in *M*. *domestica*, *D*. *melanogaster and An*. *gambiae*. Phylogenetic analysis was resolved into distinct clades among *Glossina* species, *D*. *melanogaster*, *M*. *domestica* and *An*. *gambiae* ORs [68] (Fig 7). Three paralogs of Or45a which is responds to stress in *Drosophila* larvae [69], were identified in all tsetse species (Fig 7). Expansion of Or7a and Or46a was also noted in *Glossina spp*. and *M*. *domestica* (Fig 7). A clade containing *Drosophila* cis- Vacennyl acetate receptor; Or67d homologs shows its expansion in tsetse flies. Four *Glossina* species (*G*. *austeni*, *G*. *brevipalpis*, *G*. *f*. *fuscipes and G*. *pallidipes*) had a total of five Or67d paralogs compared to six copies reported in *G*. *m*. *morsitans* [31]. Other genes that showed expansion in *Glossina* species include Or67c and Or43a (Fig 7).

**Fig 8:**
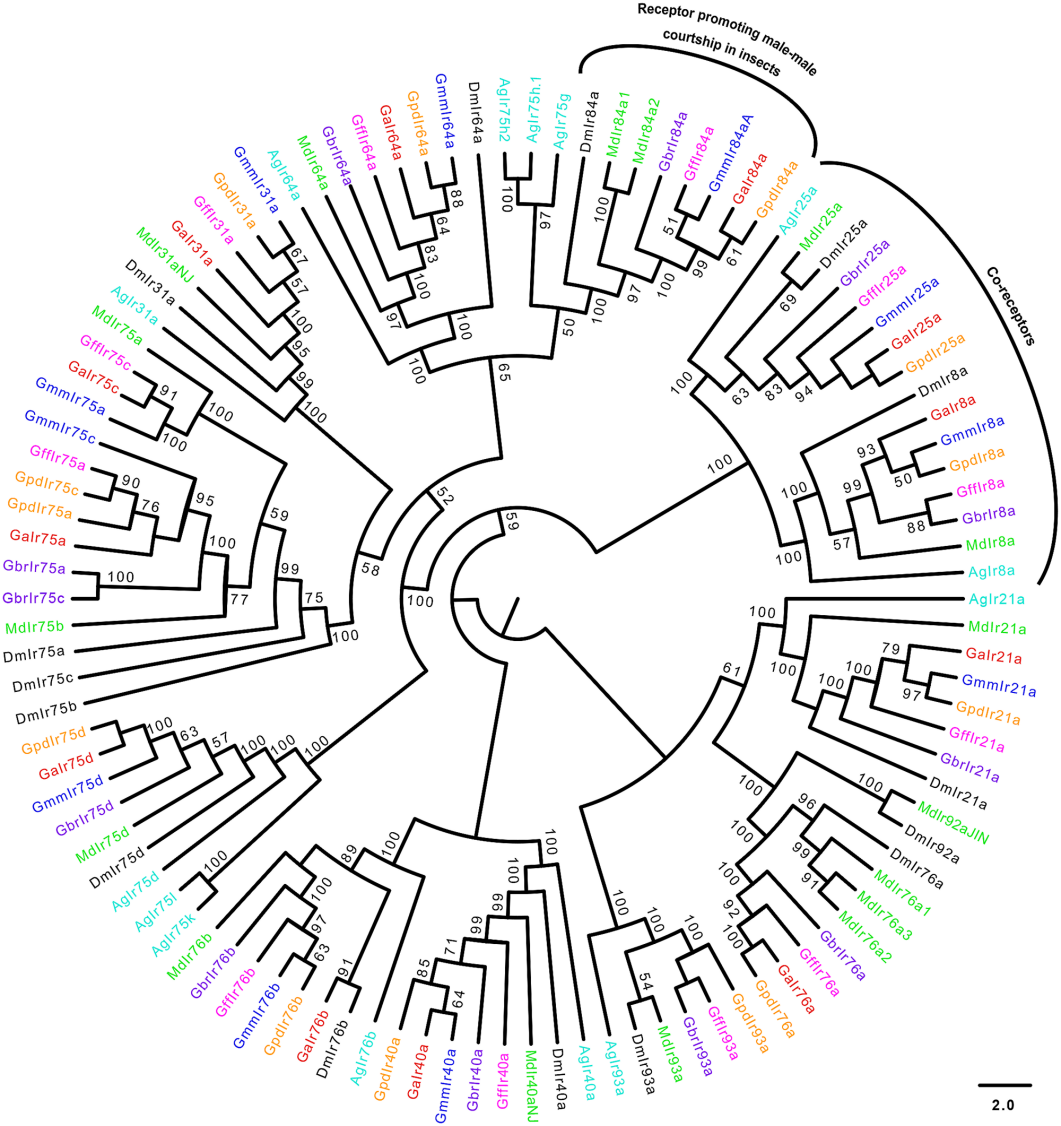
Phylogeny of antennal ionotropic receptors. Antennal IRs are primarily expressed at the antenna of the insect. Sequence alignment was performed using MuSCLE v3.8.31 and phylogeny relationship inferred using RAxML v8 with best fitting Wheelan and Goldman (WAG) model and 1000 bootstrap iterations. Different symbols and colors were used to depict IRs from the different species at the terminal nodes: *Glossina austeni* (red*), *Glossina brevipalpis* (purple*), *Glossina fuscipes fuscipes* (pink*), *Glossina morsitans morsitans* (dark blue*), *Glossina pallidipes* (light orange*), *Drosophila melanogaster* (black*), *Musca domestica* (lime green*) *and Anopheles gambiae* (sky blue*). The symbol * represents the name of the specific IR.

**Fig 9:**
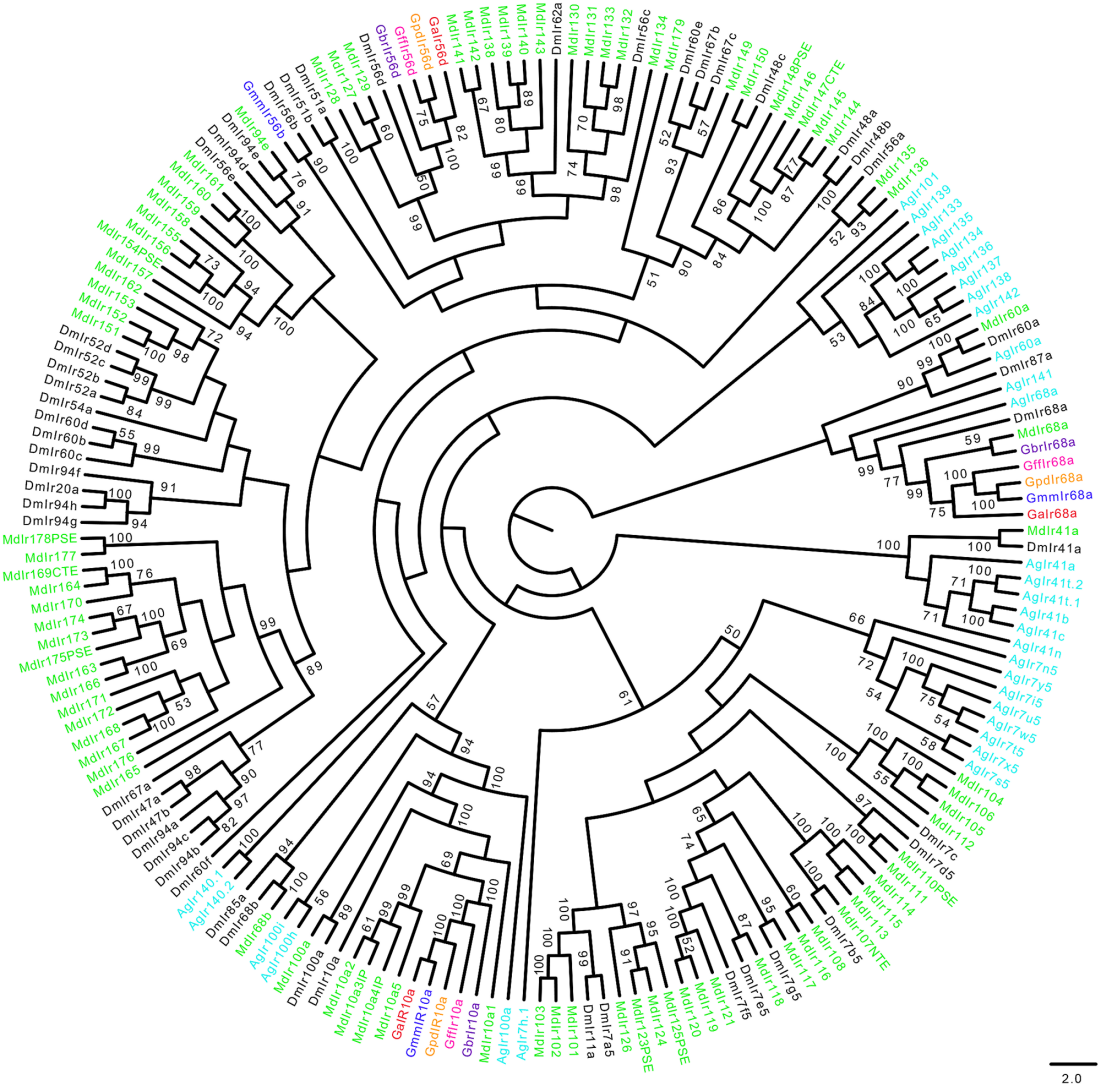
Phylogeny of divergent ionotropic receptors. Sequence alignment was performed using MuSCLE v3.8.31 and phylogeny relationship inferred using RAxML v8 with best fitting Wheelan and Goldman (WAG) model and 1000 bootstrap iterations. Different symbols and colors were used to depict IRs from the different species at the terminal nodes: *Glossina austeni* (red*), *Glossina brevipalpis* (purple*), *Glossina fuscipes fuscipes* (pink*), *Glossina morsitans morsitans* (dark blue*), *Glossina pallidipes* (light orange*), *Drosophila melanogaster* (black*), *Musca domestica* (lime green*) *and Anopheles gambiae* (sky blue*). The symbol * represents the name of the specific IR.

**Fig 10:**
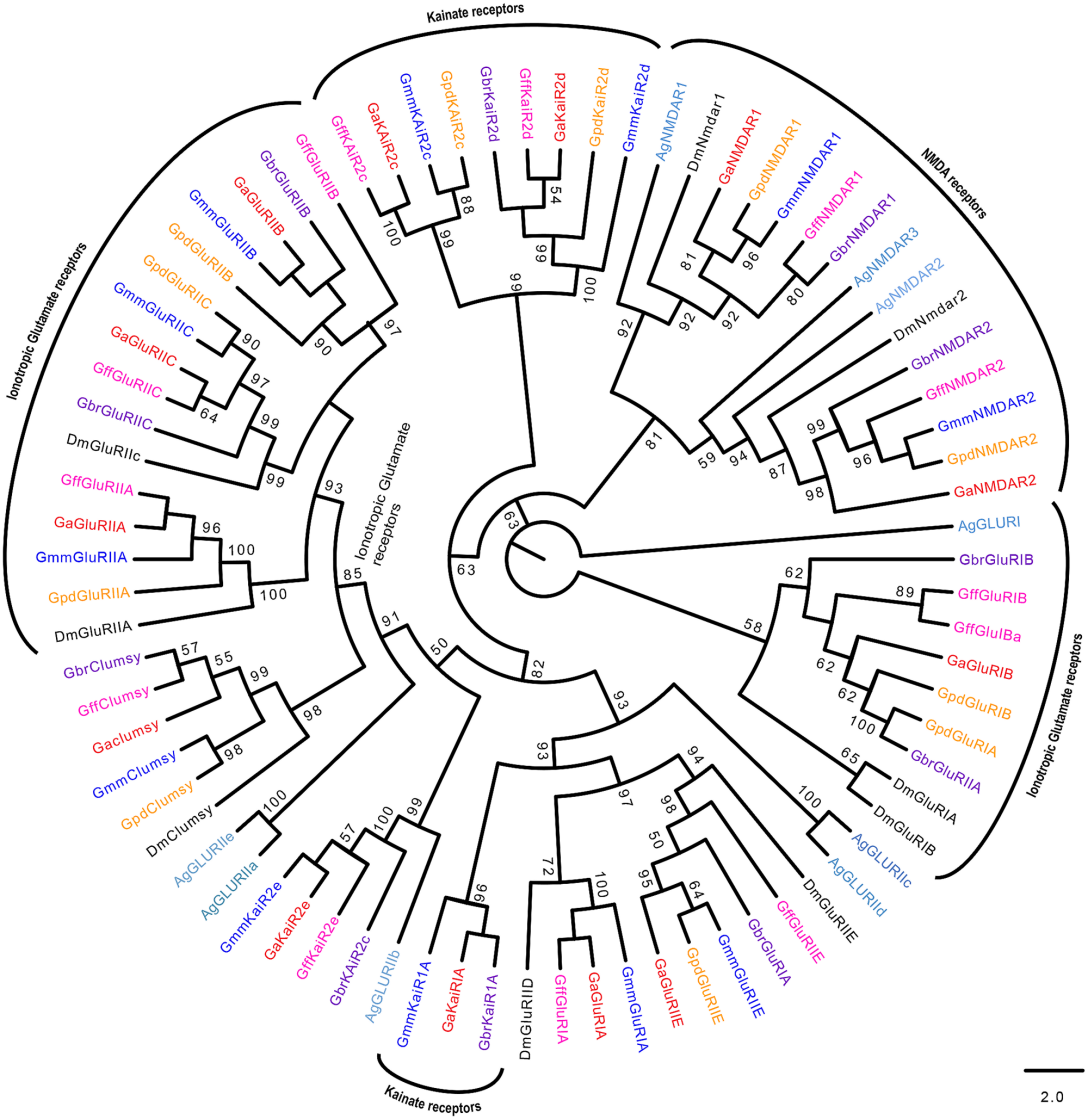
Phylogeny of ionotropic glutamate receptors and kainate receptors. Sequence alignment was performed using MuSCLE v3.8.31 and phylogeny relationship inferred using RAxML v8 with best fitting Wheelan and Goldman (WAG) model and 1000 bootstrap iterations. Different symbols and colors were used to depict IGluRs from the different species at the terminal nodes: *Glossina austeni* (red*), *Glossina brevipalpis* (purple*), *Glossina fuscipes fuscipes* (pink*), *Glossina morsitans morsitans* (dark blue*), *Glossina pallidipes* (light orange*), *Drosophila melanogaster* (black*), *Musca domestica* (lime green*) *and Anopheles gambiae* (sky blue*). The symbol * represents the name of the specific IGluR.

Similar numbers of IRs/iGluRs were identified in all tsetse species (Table 1). The homolog of a *Drosophila* Ir93a was not identified in *G*. *austeni*. Phylogeny reconstruction of IRs and iGluRs yielded highly supported clades (Figs 8–10). A total of 13 *Glossina* IR homologs clustered with their antennal *Drosophila* orthologs (Ir40a, Ir25a, Ir8aa, Ir93a, Ir21a, Ir76a, Ir76b, Ir31a, Ir75c, Ir75a, Ir75d, Ir64a and Ir84a) (Fig 8). Further, *Drosophila*-specific Ir84a and was found to have homologs in all *Glossina* species studied.

Only three of the *Glossina* predicted Irs clustered with the divergent IRs (Fig 9). These include Ir68a, Ir10a and Ir56d in *Glossina* species except *G*. *m*. *morsitans* which had a homolog of Ir56b. Although the alignment of IRs and iGluRs show similar modular arrangements (S3 and S4 Figs, respectively), iGluRs have an extra conserved arginine residue which most IRs lack. Phylogeny of the iGluRs (Fig 10) depicts their high conservation across Diptera.

### Selection Analysis

The M8 (beta & w) codeml model was found to have better representation of the data relative to M1a and M2a models, hence its adoption in calculation of LRT values. Nevertheless, some of the *d*_N_/*d*_S_ (w1M8) values were too high to be considered reliable (S1 Table); such values result from low counts of synonymous substitutions compared nonsynonymous substitutions. In addition, majority (67.02%) of the alignments were seen to have a significant p-value under the M8-M8a model. Different levels of selection were noted for majority of the intra-species paralogs (S1 Table). For instance whereas Ejbp3B showed significant selection, Ejbp3A did not show significant selection signatures. Similarly, Or45a2 and Or45a3 showed significant selection while Or45a1 did not show significant selection. Other genes with similar pattern of selection pressures are shown in S1 Table. On the contrary, only a small subset (13.64%) of gene loci was significantly identified to be under selection in the HyPhy package (S2 Table). Only four gene loci (Gr21a, Gr28b, Obp83a and GluRIIA) that were identified by the two packages could be conclusively indicated to be under selection (Table 2). Various factors such as the low number of sequences per gene loci and lack of divergence within sequences have been indicated to introduce false positives (type I error) and lack meaningful inference [70,71].

**Table 2:** Summary of four *Glossina* chemosensory gene loci identified to have signatures of positive selection. Selection analysis was performed using HyPhy package using MEME and PARRIS and compared with PAML –codeml using the M8-M8a model. lnL M8 is the likelihood of the experimental model (M8).

Gene id	lnL M8	lnL M8a	ΔLRT	p –value	w1M8	Sites by MEME	Singleton (S) /Duplicate (D)	Number of codons analyzed	ΔLRT MEME
Obp83a	-529.17	-533.174	7.943	0.0048	1.075	29	D	498	57.927
Gr21a	-1642.19	-1656.36	28.346	1.014E-7	1.18653	39	D	621	21.87
GluRIIA	-1431.52	-1387.04	8.34	0.00387	1.4264	2	S	1807	12.58
Gr28b	-1557.62	-1566.29	17.34	4.85E-5	1.75	44	D	569	6.98

lnL M8a is the likelihood of the null model (M8a), ΔLRT is the Likelihood Ratio Test = 2*(lnL M8- lnL M8a), w1M8 is the ratio of non-synonymous to synonymous mutations (*d*_N_/*d*_S_) predicted under M8 model/and p-value is the statistical measure of significance.

## Discussion

Identification and annotation of chemosensory gene families in four tsetse genomes (*G*. *austeni*, *G*. *brevipalpis*, *G*. *f*. *fuscipes* and *G*. *pallidipes*), which are representatives of all tsetse fly sub-genera, has provided a comprehensive gene repertoire necessary for undertaking comparative functional genomics.

Results of this study show a general conservation of chemosensory gene families in terms of sequence length, gene structure, and gene copy numbers across the five tsetse species. This included the previously described *G*. *morsitans morsitans* [31,24]. Specifically, high levels of conservation were observed in OBPs and CSPs; genes involved in trafficking of hydrophobic molecules across the sensillum lymph of insects [72], suggesting a safeguarded role in odorant binding. The two protein families are characterized by six and four conserved cysteine residues, respectively, with CSPs being more conserved [73]. This supports earlier observations by Sanchez-Gracia *et al*., (2009) that the CSPs family is more conserved compared to OBPs family. The majority of OBPs identified across the *Glossina* genus fall under the Classic subfamily with six conserved cysteine residues (Fig 1). This is consistent with what has been reported in genomes of related insect species such as *Drosophila* and the Mediterranean fly [14,49], suggesting that classical OBPs have conserved functions in all insects. Expansion of Obp83a (previously named Obp8-10,12 in *G*. *m*. *morsitans* [24] was noted in all tsetse species. Liu and colleagues [24] suggested that Obp83a1 could be olfactory-specific as it is expressed highly in starved females. Therefore, the expansion of Obp83a across the five tsetse species studied so far implicates its participation in host seeking, with the duplication indicating the investment made by tsetse in finding food. Co-localization of the four copies under the same scaffold (S1 Fig) suggests that they are recently duplicated paralogs that perhaps could be co-regulated. On the other hand, the presence of two *Glossina* odorant receptor paralogs (copies of Or45a and Or7a) in distantly located scaffolds may indicate the involvement of transposition in emergence. Notably, gene transposition has been reported earlier in three *Drosophila* species (*D*. *melanogaster*, *D*. *yakuba* and *D*. *simulans*) [74–76], adding credence to the occurrence of transposition as a mode of gene emergence in insects.

The complete loss of genes and/or distortion in their gene structure observed in *G*. *brevipalpis* could be attributed to evolutionary events given that it is the most ancient among the *Glossina* species studied [77]. This correlates an assumption made by Gooding and colleagues [78] who proposed that the oldest subgenus would exhibit more genetic differences, assuming a constant rate of evolution. Of two GRs (Gr32a and Gr68a), that are known to respond to pheromones, only Gr32a was present in all five *Glossina* species. This is not surprising as Gr68a also participates in sound reception [66]. Absence of Gr68a in tsetse could imply that tsetse flies rely on a different receptor other than Gr68a for sound reception, or that the insects rely entirely on their tympanal organ for this function [79]. Additionally, absence of Gr68a has been reported to reduce male-male courtship in *Drosophila* and perhaps may play the same role in tsetse flies [32]. *Glossina* IRs/iGluRs shows conservation of copy numbers. Notably, the Ir84a have homologs in all tsetse species studied here. Ir84a is a candidate receptor for phenylacetyaldehyde and has been reported to promote male courtship in *Drosophila* [80]. Presence of Ir84a in *Glossina* support male courtship to be conserved across tsetse species. On the other hand, the absence of Ir93a in *G*. *austeni* whose ligand is unknown [81] could potentially encode a defective response to either aldehydes, amines or carboxylic acids, which are primarily recognized by IRs [37].

Based on the number of chemosensory genes identified across *Glossina*, it is apparent that all tsetse fly species have a reduced chemosensory repertoire compared to *D*. *melanogaster* and *M*. *domestica*. This is in agreement with findings reported in *G*. *m morsitans* [31,42]. Noteworthy is the absence of all sugar receptors (Gr64a-f and Gr5a) in all tsetse species studied here (Fig 6). This is presumably due to the obligate hematophagous nature of both sexes in tsetse flies. Sugar receptors are present in *M*. *domestica*, *D*. *melanogaster* and *An*. *gambiae*, which feed on nectar as primary or secondary source of nutrients. Also, tsetse species lack homologs to Gr43a, which has been attributed to internal fructose sensing in *Drosophila* [67]. Gr43a mutants show an abolished preference of fructose but no difference in response to other sugars [82]. All tsetse species showed expansion of Gr21a homologs that mediates CO_2_ recognition confirming that tsetse flies are attracted to their vertebrate hosts through this volatile gas [83]. Similar to *M*. *domestica* [47], expansions of Or45a and Or67d that mediate stress response [84] and cVA reception [85], respectively, in *Drosophila*, were noted in all tsetse species. Or45a in *Drosophila* is expressed only in larvae [69] where it serves as a receptor for octyl-acetate that trigger a repellency effect [84]. Though the significance of expansion of Or45a in tsetse is yet to be understood, the receptor may potentially play roles in recognizing some undesirable cues present in tsetse’s uterus during larval development. Further, expansion of Or67d in the majority of the insect species compared in this study may point to its importance in enhancing their pheromone perception, hence mate selection [85]. Other ORs that showed expansion in tsetse include Or67c whose role is yet to be determined and Or43a, linked to benzaldehyde perception in *Drosophila* [86]. Among the annotated *Glossina* OBPs, Obp19 (a gene without homologs in *Drosophila*) was seen to have homologs in hemipterans, *Lygus lineoralis* and *Microplitis demolitor* and not in any of the close dipterans such as *M*. *domestica* or *Stomoxys calcitrans*. Moreover, Obp19 showed close phylogenetic relationship with Obp56i from all the *Glossina* species. This could imply that Obp19 is a recent paralog of Obp56i that assumes similar function to that of its homologs in hemipterans. Close phylogenetic relationship observed among *Glossina* OBPs and genes related to pheromone binding protein receptor proteins (PBPRPs) from other insects including (i.e. Obp19d, Obp28a, Obp69a, Obp83a and Obp84a) is similar to what was reported in *C*. *capitata* [87]. This implies that the role of PBRPs is well conserved in tsetse flies, as in other insect species.

Three of four gene loci (Table 2), showing strongest indication of positive selection are evolving under duplication suggesting a rapid rate in their evolution as earlier reported in ants [20] and in *Drosophila* [88]. The three genes are potentially involved in host seeking and/or taste discrimination in tsetse species and could therefore serve as targets for behavior manipulation as control measure. The Gr21a has three copies in all the five *Glossina* species and is believed to play a role in detection of CO_2_; a tsetse volatile cue from vertebrate hosts [83]. Expansion of CO_2_ receptors is also noted in the malaria vector, *An*. *gambiae* (Fig 6). This highlights the importance of CO_2_ in host location. Similar to Gr21a, Obp83a has four copies in each of the five *Glossina* species characterized so far and has been reported to be highly expressed in adult females 48 hours post feeding [24] suggesting its role in host finding. The only singleton found to be under significant selection is GluRIIA, but its role in tsetse is unknown. However, the homolog of GluRIIA in *Drosophila* has been implicated in postsynaptic signaling at the neuromuscular junction [89]. Though few genes were found to harbor signatures of natural selection, it is evident that those identified are inclined towards host seeking and perhaps are responsible for diverse host preference observed across different species [13,90,91]. The discrepancy in the number of gene loci identified to be under positive selection by PAML and HyPhy package could be due to few sequences available for the analysis. In addition to forces of natural selection, the observed behavioral differences exhibited by tsetse species could be as a result of unraveled diversity in their signal transduction machinery and/or post translational modification in their respective chemosensory proteins. Two different odor transduction mechanisms have been proposed in insects [92]. They include (I) the receptor-mediated (ion-channel) mechanism which does not rely on G-protein signaling pathway [93] and (II) the G-protein cascade approach in which binding of semiochemicals to ORs is thought to activate the cyclic-nucleotide pathway [94,95]. To date, little is known about the interaction between the tsetse’s specific ORs and their corresponding ligands and their downstream processing in the fly’s central nervous system (CNS). Receptor-ligand interaction marks the beginning of odor processing that leads to a behavioral response. Post-translational modification is known to permit change of the amino acid properties as a reaction towards physiological needs of an organism [96]. For instance, phosphorylation has been attributed to elasticity of ion channels involved in signaling [97]. Thus, it is important to study the downstream processes involved in odor processing across tsetse species to identify any underlying differences responsible for their behavior towards hosts. Additionally, tsetse species may have developed an adaptation to specific odours based on learning. This type of learning has been reported to influence host selection in tsetse [91]. It is therefore possible that learning could play a role in differentially recognizing odours observed across different tsetse species.

In general, tsetse species have a conserved chemosensory gene repertoire with genes sparsely distributed across their genomes. This study did not find significant gene loss/gain between species, except *G*. *brevipalpis*, the presumed ancestral species. A few of the chemosensory genes in tsetse are rapidly evolving through duplication and among them, genes potentially associated with host finding are under strong positive selection pressure, presumably to confer adaptation to host odours. These genes among others could form potential molecular targets for control. The power to detect genes under natural selection and its influence on shaping olfaction in tsetse flies was limited by the number of sequences available. More gene sequences may yield better results in future. This study highlights the need to undertake functional studies on chemosensory genes of tsetse and to study the down-stream odor signaling pathway to enhance our understanding on differential behavior observed across tsetse species and how it can be used in improving current control strategies. Knowledge of differential host responses of sympatric tsetse species will aid in development of an integrated universal and cost-effective control strategy for vectors of trypanosomiasis.

## Supporting Information

S1 Dataset Metadata for genes annotated in four species of *Glossina*. Metadata for each protein chemosensory family is contained in a separate sheet. CSPs—sheet 1, SNMPs—sheet 2, GRs –sheet3, OBPs –sheet 4, ORs –sheet 5 and IRs –sheet 6. For every sequence, the following data is provided in columns A-G of each sheet: Gene name, VectorBase identifier, scaffold where it is located, number of exons, strand orientation, length of the amino acid sequence and the associated scaffold coordinates.
(XLS)

S2 Dataset Alignment files of the amino acid sequences. Multiple sequence alignments of five *Glossina* species, *Drosophila melanogaster*, *Anopheles gambaie*, *Musca domestica* and *Ceratitis capitata* used in construction of phylogenies of the chemosensory gene.
(ZIP)

S1 Fig VectorBase web Apollo screenshots. Screenshots illustrating gene structure and tandem arrangement of selected chemosensory genes. Four copies of Obp83a (part A) thought to be olfactory specific in *Glossina*Two Or7a homologs (part B) and two Or56a homologs (part C).
(PDF)

S2 Fig Multiple alignment of Obp19 and Obp56i from *Glossina*, Obp16-20 from *M*. *domestica* and Obp56i from *D*. *melanogaster*. Variation of amino acids between conserved cysteine(s) C3 and C4 in Obp56i and Obp19 from *Glossina*. Their homologs in *M*. *domestica* and *D*. *melanogaster* appear more conserved around the same region.
(PDF)

S3 Fig Alignment of IRs amino acid sequences showing conserved residues that constitute the pore region. (PDF)

S4 Fig Alignment of IGluRs amino acid sequences showing conserved residues that constitute the pore region. (PDF)

S1 Table *Glossina* chemosensory gene loci identified to have signatures by PAML analysis using the M8-M8a model. lnL M8 is the likelihood of the experimental model (M8), lnL M8a is the likelihood of the null model (M8a), ΔLRT is the Likelihood Ratio Test = 2*(lnL M8- lnL M8a), w1M8 is the ratio of Non-synonymous to synonymous mutations (*d*_N_/*d*_S_) predicted under M8 model/and p-value is the statistical measure of significance.
(PDF)

S2 Table Tsetse chemosensory genes identified as having signatures of positive selection by codon-based alignment methods MEME and PARRIS in Datamonkey analysis. (PDF)
