# Correction: Genome-Wide Comparative Analysis of Chemosensory Gene Families in Five Tsetse Fly Species

**DOI:** 10.1371/journal.pntd.0005199

**Published:** 2016-12-05

**Authors:** Rosaline Macharia, Paul Mireji, Edwin Murungi, Grace Murilla, Alan Christoffels, Serap Aksoy, Daniel Masiga

There are errors in the accession numbers for *Glossina morsitans morsitans* in the fourth paragraph under the subheading “Odorant Binding Proteins” in the Methods section. It should read as follows:

GMOY000890-PA, GMOY002825-PA,GMOY002826-PA, GMOY007757-PA, GMOY006521-PA, GMOY006522-PA, GMOY009708 -PA,GMOY005548 -PA,GMOY004317 -PA,GMOY004316 -PA,GMOY005184 -PA,GMOY012275 -PA,GMOY005550 -PA,GMOY005184 -PA,GMOY002859-PA,GMOY006523-PA

The list of accession numbers for *Glossina morsitans morsitans* in the fourth paragraph under the subheading “Odorant Receptors” in the Methods section is incomplete. It should read as follows:

GMOY005610 -PA,GMOY005796 -PA, GMOY004772 -PA,GMOY012018 -PA,GMOY009475 -PA,GMOY010761 -PA,GMOY009271 -PA,GMOY003312 -PA,GMOY001365 -PA,GMOY005386 -PA,GMOY012322 -PA,GMOY011399 -PA,GMOY010839 -PA,GMOY012357 -PA,GMOY008038 -PA,GMOY005084 -PA,GMOY005479 -PA,GMOY011902 -PA,GMOY004392 -PA,GMOY012356 -PA,GMOY006480 -PA,GMOY006479-PA,GMOY006265- GMOY012218-PA, GMOY001927-PA, GMOY012357-PA, GMOY012253-PA
