# Supplementary figures and images for: Genome-Wide Comparative Analysis of Chemosensory Gene Families in Five Tsetse Fly Species
Source: PLoS Negl Trop Dis. 2016 Feb 17;10(2):e0004421. doi: 10.1371/journal.pntd.0004421 (PMC4757090; doi:10.1371/journal.pntd.0004421)

**(A): - OBP83a homologs**

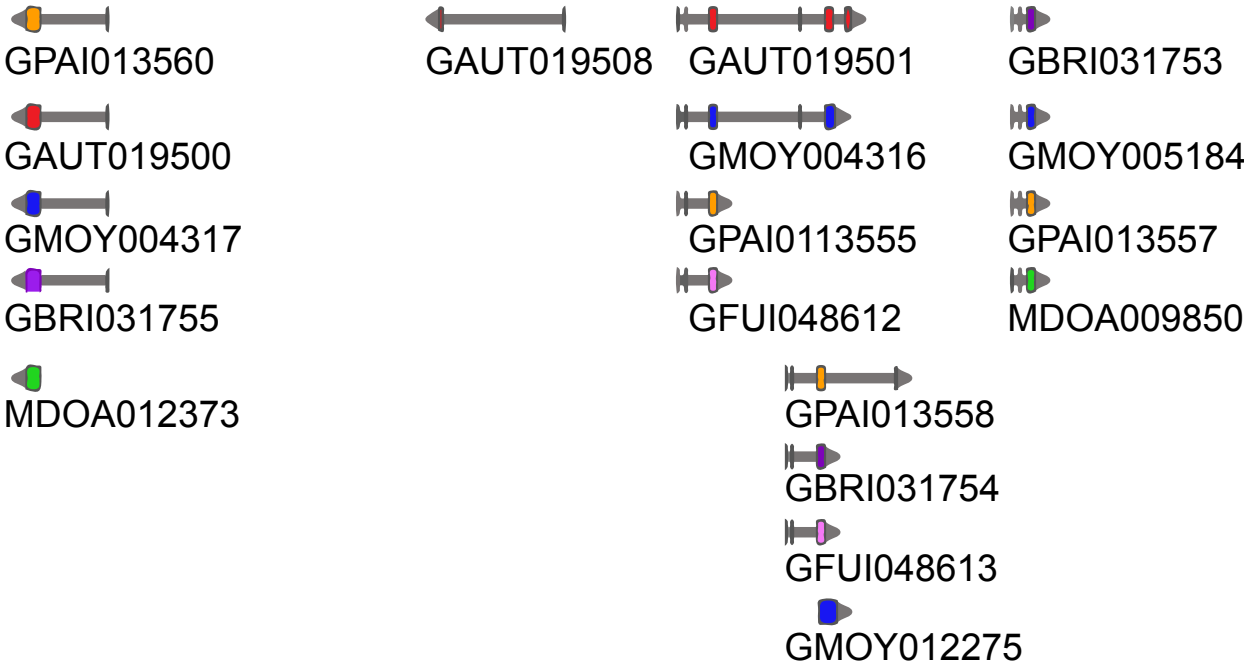

**(B): - OR7a homologs**

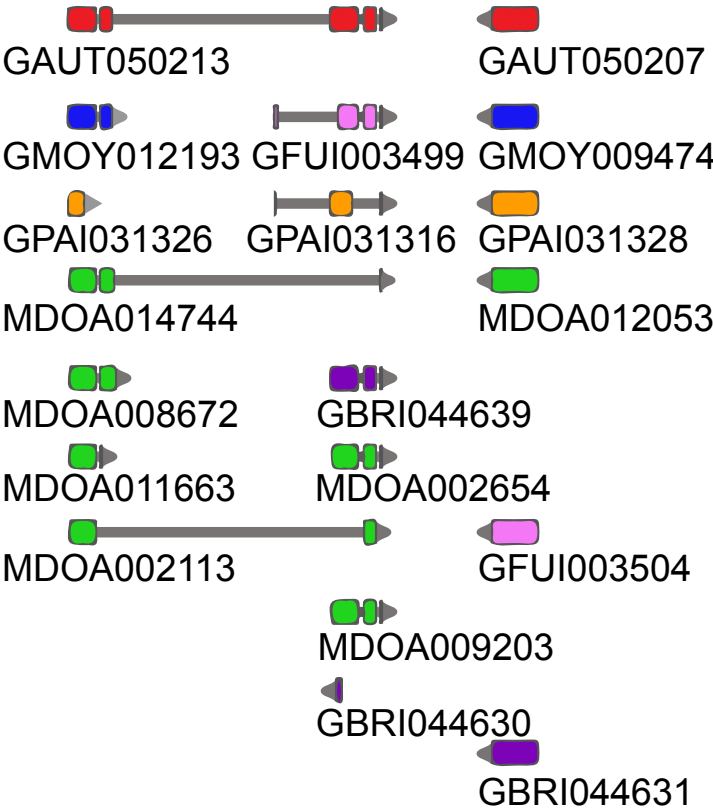

**(C): - OR56a homologs**

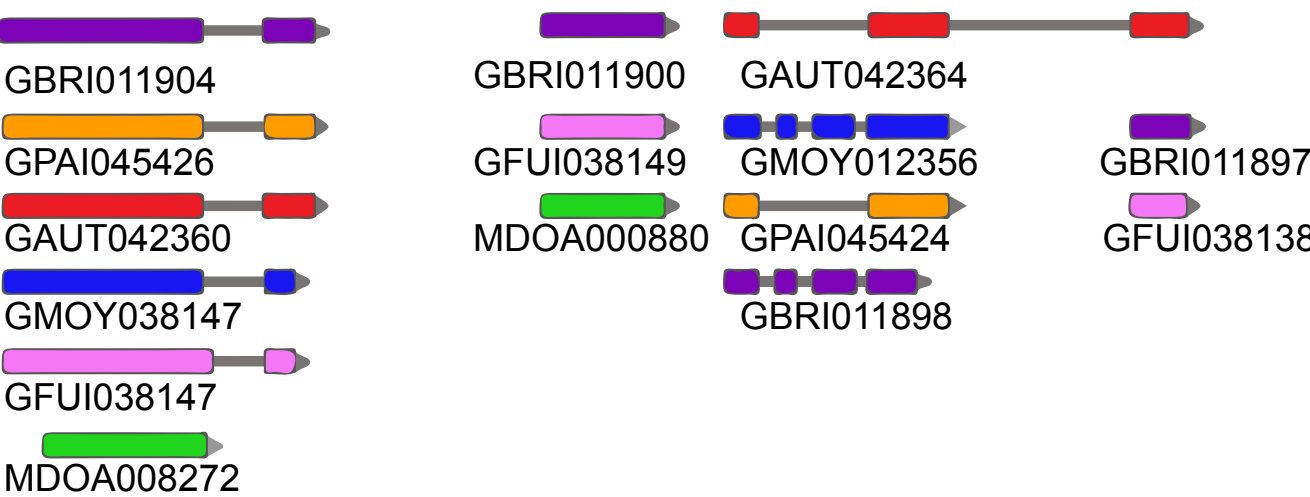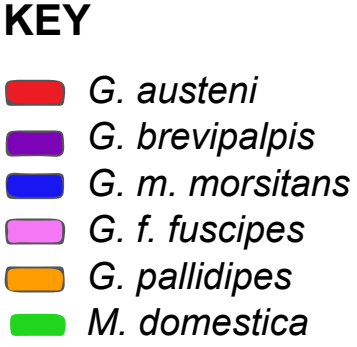

Supplement: S1 Fig — Screenshots illustrating gene structure and tandem arrangement of selected chemosensory genes. Four copies of Obp83a (part A) thought to be olfactory specific in GlossinaTwo Or7a homologs (part B) and two Or56a homologs (part C). (PDF) [file pntd.0004421.s003.pdf]

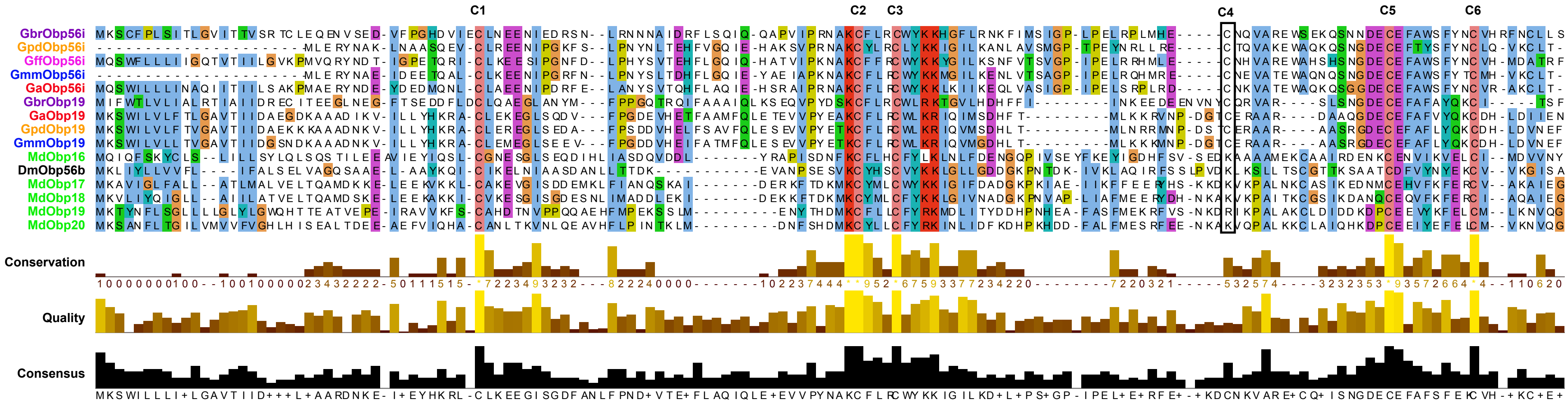

Supplement: S2 Fig — Variation of amino acids between conserved cysteine(s) C3 and C4 in Obp56i and Obp19 from Glossina. Their homologs in M. domestica and D. melanogaster appear more conserved around the same region. (PDF) [file pntd.0004421.s004.pdf]
