# Supplementary material for: Genome-Wide Comparative Analysis of Chemosensory Gene Families in Five Tsetse Fly Species
Source: PLoS Negl Trop Dis. 2016 Feb 17;10(2):e0004421. doi: 10.1371/journal.pntd.0004421 (PMC4757090; doi:10.1371/journal.pntd.0004421)
Supplement: S3 Fig — (PDF) [file pntd.0004421.s005.pdf]

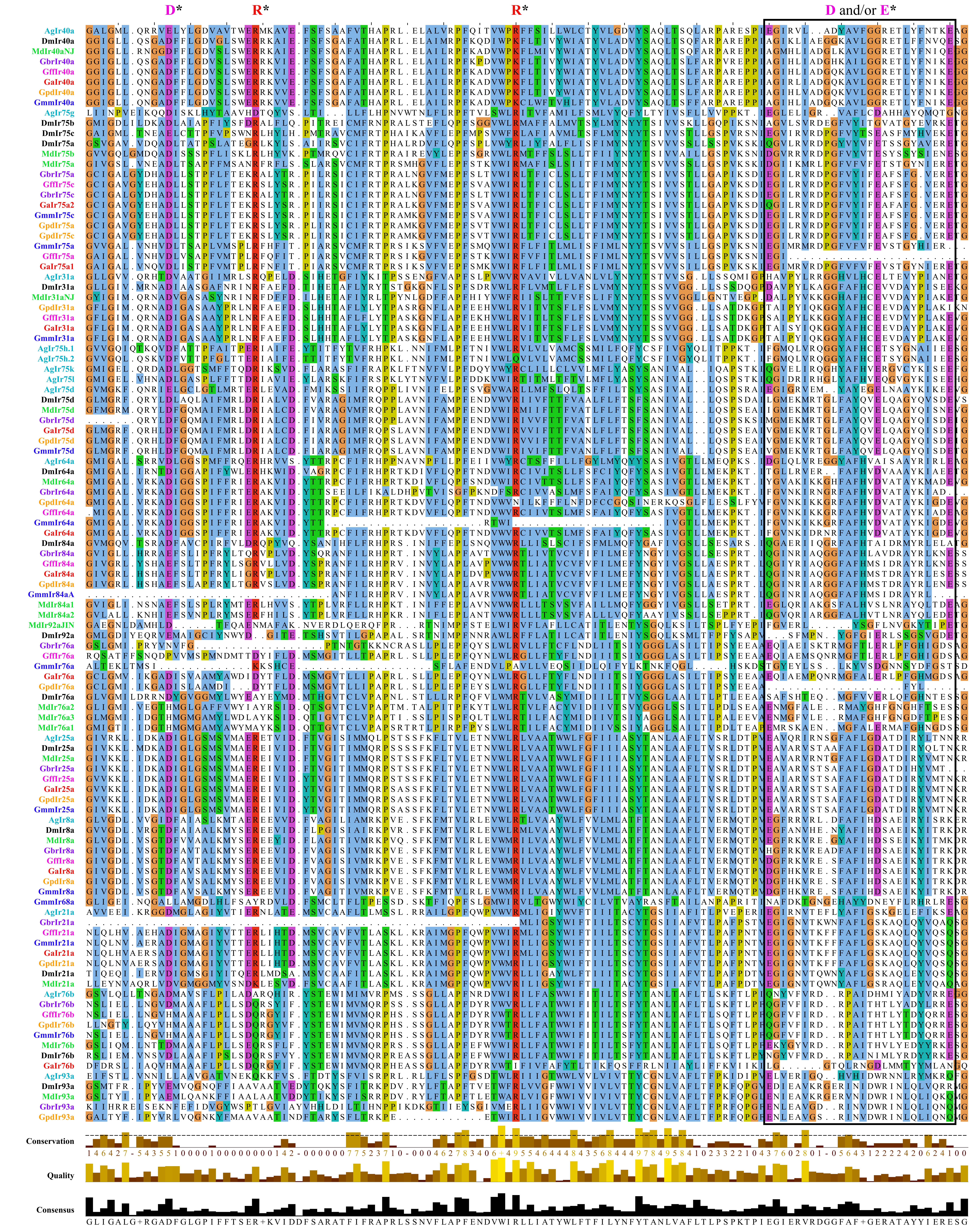
D*
R*
R*
D and/or E*
Conservation
Quality
Consensus
