# Supplementary material for: Genome-Wide Comparative Analysis of Chemosensory Gene Families in Five Tsetse Fly Species
Source: PLoS Negl Trop Dis. 2016 Feb 17;10(2):e0004421. doi: 10.1371/journal.pntd.0004421 (PMC4757090; doi:10.1371/journal.pntd.0004421)
Supplement: S4 Fig — (PDF) [file pntd.0004421.s006.pdf]

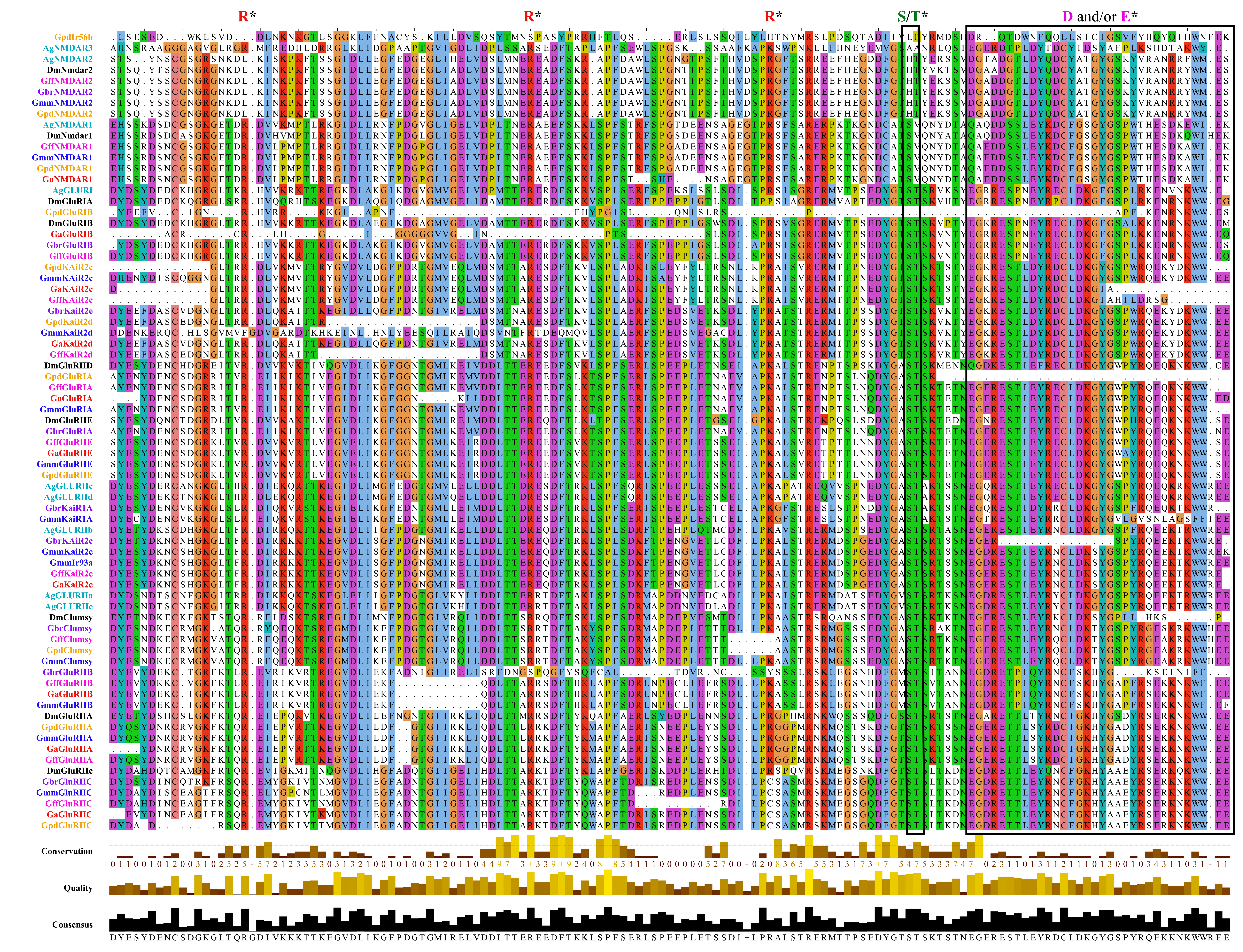
R*
R*
R*
S/T*
D and/or E*
GpdIr56b
AgNMDAR3
AgNMDAR2
DmNmdar2
GffNMDAR2
GbrNMDAR2
GmmNMDAR2
GpdNMDAR2
AgNMDAR1
DmNmdar1
GffNMDAR1
GmmNMDAR1
GpdNMDAR1
GaNMDAR1
AgGLURI
DmGluRIA
GpdGluRIB
DmGluRIB
GaGluRIB
GbrGluRIB
GffGluRIB
GpdKAiR2c
GmmKAiR2c
GaKAiR2c
GffKAiR2c
GbrKaiR2e
GpdKaiR2d
GmmKaiR2d
GaKaiR2d
GffKaiR2d
DmGluRIID
GpdGluRIA
GffGluRIA
GaGluRIA
GmmGluRIA
DmGluRIIE
GbrGluRIA
GffGluRIIE
GaGluRIIE
GmmGluRIIE
GpdGluRIIE
AgGLURIIc
AgGLURIId
GbrKaiR1A
GmmKaiR1A
AgGLURIIb
GbrKAiR2c
GmmKaiR2e
GmmIr93a
GffKaiR2e
GaKaiR2e
AgGLURIIa
AgGLURIIe
DmClumsy
GbrClumsy
GffClumsy
GpdClumsy
GmmClumsy
GbrGluRIIB
GffGluRIIB
GaGluRIIB
GmmGluRIIB
DmGluRIIA
GpdGluRIIA
GmmGluRIIA
GaGluRIIA
GffGluRIIA
DmGluRIIc
GbrGluRIIC
GmmGluRIIC
GffGluRIIC
GaGluRIIC
GpdGluRIIC
Conservation
Quality
Consensus
DYESYDENCSDGKGLTQRGDIVKKKTTKEGVDLIKGFPDGTGMIRELVDDLTTEREEDFTKKLSPFSERLSPEEPLETSSDI+LPRALSTRERMTTPSEDYGTSTSKTSTNEGERESTLEYRDCLDKGYGSPYRQEKKNKWREE
