# Supplementary material for: Genome-Wide Comparative Analysis of Chemosensory Gene Families in Five Tsetse Fly Species
Source: PLoS Negl Trop Dis. 2016 Feb 17;10(2):e0004421. doi: 10.1371/journal.pntd.0004421 (PMC4757090; doi:10.1371/journal.pntd.0004421)
Supplement: S1 Table — lnL M8 is the likelihood of the experimental model (M8), lnL M8a is the likelihood of the null model (M8a), ΔLRT is the Likelihood Ratio Test = 2*(lnL M8- lnL M8a), w1M8 is the ratio of Non-synonymous to synonymous mutations (dN/dS) predicted under M8 model/and p-value is the statistical measure of significance. (PDF) [file pntd.0004421.s007.pdf]

Gene Id	ln M8	ln M8a	Δln (M8-M8a)	ΔLRT	p-value	w1M8
A10p	-811.159168	-817.800546	6.641378	13.282756	0.000267858	2.29248
CSP1	-382.682564	-385.27889	2.596326	5.192652	0.02268258	0.0001
ejbp3A	-696.258521	-696.304455	0.045934	0.091868	0.761815678	1.68715
ejbp3B	-518.928289	-523.260899	4.33261	8.66522	0.003243409	0.59149
GluRIA	-1779.752613	-1786.335007	6.582394	13.164788	0.000285259	1.7262
GluRIB	-674.078481	-674.078482	1E-06	2E-06	0.998871621	1.70965
GluRIIA	-1387.04268	-1391.213979	4.171299	8.342598	0.003872618	1.42643
GluRIIC	-2667.323657	-2671.053334	3.729677	7.459354	0.006310766	2.72147
GluRIIE	-3028.805848	-3028.093265	-0.712583	-1.425166	-	1.57715
Gr21a1	-1591.535275	-1605.840088	14.304813	28.609626	8.85409E-08	1.30195
Gr21a2	-2064.268263	-2080.9993	16.731037	33.462074	7.26673E-09	1.01281
Gr21a3	-1270.762992	-1282.246557	11.483565	22.96713	1.64795E-06	1.24483
Gr28B	-1526.110898	-1533.825774	7.714876	15.429752	8.56295E-05	1.36457
Gr28C	-1589.121628	-1598.749933	9.628305	19.25661	1.14274E-05	2.13749
Gr33a	-1261.766144	-1262.140791	0.374647	0.749294	0.386699848	1.68448
Gr58c	-1507.540911	-1514.059466	6.518555	13.03711	0.000305379	2.10371
Gr59f1	-1815.566077	-1815.791053	0.224976	0.449952	0.502357751	1.82731
Gr59f2	-2010.700595	-2011.986454	1.285859	2.571718	0.108789555	1.83603
Gr63a	-1444.402808	-1455.818486	11.415678	22.831356	1.76857E-06	1.83256
Gr66a	-1183.440173	-1187.896531	4.456358	8.912716	0.002831918	2.05806
ir10a	-2392.593054	-2396.646383	4.053329	8.106658	0.004410297	1.37199
ir21a	-2199.702085	-2208.512353	8.810268	17.620536	2.69661E-05	1.15853
ir25	-3264.733748	-3275.303169	10.569421	21.138842	4.27181E-06	1.50841
ir31a	-2508.134615	-2521.610983	13.476368	26.952736	2.08492E-07	1.55333
ir40a	-3421.099018	-3431.515305	10.416287	20.832574	5.01232E-06	1.88823
ir56b	-1496.79393	-1502.338468	5.544538	11.089076	0.000868377	1.86781
ir64a	-916.914224	-923.116909	6.202685	12.40537	0.000428101	1.31106
ir68a	-2046.262936	-2054.194127	7.931191	15.862382	6.81194E-05	2.02104
ir75a	-104.160337	-106.330077	2.16974	4.33948	0.037238302	2.01713
ir75d	-1212.436183	-1217.669923	5.23374	10.46748	0.001214943	2.00941
ir76a	-1037.475269	-1040.520959	3.04569	6.09138	0.013584296	1.33976
ir76b	-1963.879283	-1981.541563	17.66228	35.32456	2.7909E-09	1.95194
ir84a	-2557.239819	-2562.557501	5.317682	10.635364	0.001109454	1.72142
KaiR2c	-2017.042121	-2028.777698	11.735577	23.471154	1.26801E-06	1.57074
KaiR2d	-2530.319767	-2536.90377	6.584003	13.168006	0.00028477	1.60019
KaiR2e	-4564.736913	-4617.805034	53.068121	106.136242	6.88361E-25	1.67408
NMDAR1	-1433.452954	-1443.795972	10.343018	20.686036	5.41092E-06	1.32275
NMDAR2	-1525.774413	-1525.774787	0.000374	0.000748	0.978180906	999

Gene Id	ln M8	ln M8a	Δln(M8-M8a)	ΔLRT	p-value	w1M8
Obp19c	-592.597893	-594.309115	1.711222	3.422444	0.064315745	1.83436
Obp19d	-436.845096	-439.06433	2.219234	4.438468	0.03513777	1.00041
Obp28a	-191.907338	-196.96203	5.054692	10.109384	0.001475163	0.76343
Obp56d	-1065.703464	-1066.119903	0.416439	0.832878	0.361441647	2.09463
Obp56e2	-602.066258	-603.426361	1.360103	2.720206	0.099085258	1.74658
Obp56h	-722.263364	-724.565057	2.301693	4.603386	0.03190888	1.38401
Obp57c	-510.380555	-511.77078	1.390225	2.78045	0.09542138	1.28414
Obp69a	-361.75084	-361.750075	-0.000765	-0.00153		1.80803
Obp83a1	-969.07813	-970.491354	1.413224	2.826448	0.092723254	1.21639
Obp83a2	-489.760434	-498.019898	8.259464	16.518928	4.81668E-05	0.54851
Obp83a3	-322.039075	-327.242169	5.203094	10.406188	0.001255937	0.69415
Obp83a4	-335.818905	-336.829961	1.011056	2.022112	0.155023445	1.84241
Obp83cd	-1233.336032	-1234.506778	1.170746	2.341492	0.12596889	1.48651
Obp83ef	-1027.819822	-1029.299338	1.479516	2.959032	0.08539908	1.71375
Obp83g	-677.0205	-682.371204	5.350704	10.701408	0.00107054	1.7544
Obp84a	-479.832141	-479.832141	0	0	1	1.63039
Obp8a	-529.980427	-532.186649	2.206222	4.412444	0.035677697	1.31538
Obp99b	-618.505767	-620.759341	2.253574	4.507148	0.033753482	1.90399
Obp99c	-554.783115	-559.879799	5.096684	10.193368	0.001409467	0.36278
Obp99d	-917.796276	-918.187654	0.391378	0.782756	0.376299582	1.38129
or13a	-1612.489811	-1619.172167	6.682356	13.364712	0.000256403	1.4792
or19b	-1610.528045	-1613.28506	2.757015	5.51403	0.018864533	1.93225
or24a	-1921.263337	-1921.686614	0.423277	0.846554	0.35752903	1.99768
or2a	-2470.057777	-2476.275713	6.217936	12.435872	0.000421166	2.0718
or42b	-958.367761	-968.196179	9.828418	19.656836	9.26712E-06	1.0048
or43a1	-1163.982537	-1166.254384	2.271847	4.543694	0.033040222	2.41268
or43a2	-1088.231672	-1088.231672	0	0	1	1.8918
or45a1	-1762.777159	-1763.196956	0.419797	0.839594	0.359512916	1.73313
or45a2	-1109.761898	-1124.325497	14.563599	29.127198	6.77788E-08	0.95888
or45a3	-1866.857618	-1872.732158	5.87454	11.74908	0.000608733	1.35203
or46a1	-2398.70866	-2403.760216	5.051556	10.103112	0.001480192	1.46483
or46a	-2458.908629	-2462.717437	3.808808	7.617616	0.005780085	1.48063
or47b	-1487.100413	-1488.556496	1.456083	2.912166	0.087913748	1.57852
or49b	-796.40015	-799.609305	3.209155	6.41831	0.011294961	0.75688
or56a	-232.568085	-232.568085	0	0	1	1.83446
or59a	-1749.879123	-1757.103331	7.224208	14.448416	0.000144051	1.80968
or63a	-525.829913	-525.829913	0	0	1	2.62606
or67a	-1373.083959	-1379.531518	6.447559	12.895118	0.00032944	0.87018

Gene Id	ln M8 (L1)	ln M8a (Ln1)	Δln(M8-M8a)	ΔLRT	p-value	w1M8
or67d5	-1952.938323	-1961.108488	8.170165	16.34033	5.29255E-05	1.51376
or67d6	-1543.942602	-1547.266569	3.323967	6.647934	0.009927087	1.24351
or67d2	-1069.648332	-1069.648332	0	0	1	1.23977
or7a1	-2608.22251	-2622.52591	14.3034	28.6068	8.86703E-08	1.51628
or7a2	-2425.510498	-2425.573017	0.062519	0.125038	0.723633334	1.01809
or82a	-1523.492611	-1535.596667	12.104056	24.208112	8.64673E-07	1.43647
or85b	-1561.679373	-1565.079367	3.399994	6.799988	0.009115849	1.3
or85d	-1894.523481	-1896.810886	2.287405	4.57481	0.032445348	1.52842
or85e	-2015.021557	-2019.404637	4.38308	8.76616	0.003068709	1.31723
or88a	-1277.767544	-1283.397827	5.630283	11.260566	0.000791711	1.13272
Orco	-608.602208	-616.587685	7.985477	15.970954	6.43218E-05	0.93643
PhekIII	-539.535291	-539.976337	0.441046	0.882092	0.347629342	1.81391
snmp1	-860.156544	-876.02422	15.867676	31.735352	1.76677E-08	0.76972
snmp2	-824.334696	-826.228048	1.893352	3.786704	0.051661294	3.56878
