# Supplementary material for: Genome-Wide Comparative Analysis of Chemosensory Gene Families in Five Tsetse Fly Species
Source: PLoS Negl Trop Dis. 2016 Feb 17;10(2):e0004421. doi: 10.1371/journal.pntd.0004421 (PMC4757090; doi:10.1371/journal.pntd.0004421)
Supplement: S2 Table — (PDF) [file pntd.0004421.s008.pdf]

Gene id	Singleton (S) or Duplicate (D)	Number of Codons Analysed	ΔLRT = 2×(lnL1 – lnL0)	P-value estimated by PARRIS at p<0.05	Number of Sites by MEME at p<0.05
Gr21a	D	621	21.8705	0	39
Gr28b	D	569	6.97557	0.000867529	41
Gr59f	D	669	27.0006	0	39
Obp19a	S	203	6.98971	0.0303513	6
Obp56e	D	273	7.4433	0.0237864	11
Obp56i	S	367	6.41513	0.0404517	6
Obp57c	S	199	9.91644	0.00702317	1
Obp69a	S	178	7.34839	0.0253618	2
Obp83a	D	498	57.9273	2.63789E-13	29
Or45a	D	601	13.3874	0	47
Or67d	D	606	40.9955	0	131
GluRIIA	S	1807	12.5879	0.0177759	2
